# Decoupling the electronic and geometric effects of Pt catalysts in selective hydrogenation reaction

**DOI:** 10.1038/s41467-022-31313-4

**Published:** 2022-06-21

**Authors:** Zhe Wang, Chunpeng Wang, Shanjun Mao, Bing Lu, Yuzhuo Chen, Xie Zhang, Zhirong Chen, Yong Wang

**Affiliations:** 1 Advanced Materials and Catalysis Group, Center of Chemistry for Frontier Technologies, State Key Laboratory of Clean Energy Utilization, Institute of Catalysis, Department of Chemistry, Zhejiang University, Hangzhou, 310028 People’s Republic of China; 2 College of Chemical and Biological Engineering, Zhejiang University, Hangzhou, 310028 People’s Republic of China

**Keywords:** Heterogeneous catalysis, Sustainability, Catalytic mechanisms

## Abstract

Decoupling the electronic and geometric effects has been a long cherished goal for heterogeneous catalysis due to their tangled relationship. Here, a novel orthogonal decomposition method is firstly proposed to settle this issue in *p*-chloronitrobenzene hydrogenation reaction on size- and shape-controlled Pt nanoparticles (NPs) carried on various supports. Results suggest Fermi levels of catalysts can be modulated by supports with varied work function (*W*_f_). And the selectivity on Pt NPs of similar size and shape is linearly related with the *W*_f_ of support. Optimized Fermi levels of the catalysts with large *W*_f_ weaken the ability of Pt NPs to fill valence electrons into the antibonding orbital of C–Cl bond, finally suppressing the hydrodehalogenation side reaction. Foremost, the geometric effect is firstly spun off through orthogonal relation based on series of linear relationships over various sizes of Pt NPs reflecting the electronic effect. Moreover, separable nested double coordinate system is established to quantitatively evaluate the two effects.

## Abstract (web summary)

Decoupling the electronic and geometric effects has been a long cherished goal for heterogeneous catalysis due to their tangled relationship. Here the authors propose a novel orthogonal decomposition method to decouple the electronic and geometric effect for supported metal catalysts.

## Introduction

The structure-performance relationship has been one of the core topics since the emergence of catalysis^1–3^. In terms of the active components for supported metal catalysts, the structure-performance relationship is mainly reflected by the electronic and geometric effects^4–6^. However, for structure-sensitive reactions, it is not easy to decouple them and correlate them with the catalytic performance respectively due to the complex structure of the active sites and the involved electronic metal-support interaction (EMSI)^7–9^. Besides the surface science research^10^, the most common experimental approach at present is size and morphology regulation based on the Wulff’s construction rule^11^, involving in the hydrogenation, oxidation, coupling, and other reactions^7,12^. Along with the decrease of the metal nanoparticle (NP) size, the proportion of edge and corner atoms increases smoothly at first and then rises sharply when the size is less than 5 nm, which means that the coordination environment of NPs will change dramatically in this size range and significantly alter the catalytic performance^13^. However, reports have shown that the orbital energy level of metal NPs will also split along with the size range, even tends to be the discrete molecular orbitals^14,15^. In addition, the regulation radius on charge modulation of the metal particles by supports is also limited^16^. All these indicate that the electronic effect is not uniform with the size of metal NPs, and the strong coupling between electronic and geometric effects makes this matter unresolved till now.

For instance, selective hydrogenation of halonitrobenzenes (HNBs) to haloanilines (HANs), a vital process in the production of organic intermediates like pharmaceuticals, herbicides, dyes, etc, suffers from severe selectivity loss of HANs due to the hydrogenolysis of the carbon-halogen bonds, especially at high conversions^17–19^. Choosing appropriate supports to regulate the electronic structure of metal NPs and promote the selectivity of this reaction for supported Pt catalyst have been substantiated to be highly desirable^18,20^. Citing as an example, in the hydrogenation of *p*-chloronitrobenzene (*p*-CNB), research showed that the electron-rich Pt NPs on nitrogen-doped carbon nanotubes should account for the enhanced selectivity^21^. However, fully suppressed hydrodechlorination was also observed over higher oxidation state of Pt over iron oxide^22,23^. Obviously, despite all these efforts, the effect of electronic properties of Pt NPs on the reaction selectivity still remain controversial. In fact, the dehalogenation of HNBs or HANs has been reported to be sensitive to the size and shape of Pt NPs^24–26^, which were not considered in the above studies. Recently, selective poisoning or deposition method offered an alternative^27^, but still faced the accuracy problem and the support effect from deposit sediments. The rapid development of size- and shape-controlled colloidal nanoparticles provided us a great chance to well resolve the issue after well confirmation in other size- or shape-sensitive reaction^28,29^.

In the scope of our knowledge, two main puzzles should be handled to address the above issue. Firstly, find an appropriate experimental physicochemical parameter that can reflect the change of electronic structure and be correlated with the catalytic performance. Secondly, identify the relationship between the state function of electronic structure and the changed size or morphology of metal NPs. Herein, through size- and shape-controlling methods and carefully selected mild surfactant-removing strategy, a series of supported Pt catalysts with similar particle size and shape were obtained over various supports to systematically investigate the electronic and geometric effect in *p*-CNB hydrogenation. A novel orthogonal decomposition method is firstly proposed and employed to successfully decouple the electronic and geometric effects in this reaction. Results indicated that the degree of electron transfer between Pt NPs of similar size and shape and supports was positively correlated with the work function (*W*_f_) of support, and the distinctions of Fermi levels on supported Pt NPs had significant impacts on the cleavage of the C-Cl bond, which eventually determined the selectivity of *p*-CAN. Based on a series of linear scaling relationships reflecting the electronic effect, the geometric effect was successfully spun off through the orthogonal relation. Separable nested double coordinate system was then established to quantitatively evaluate the two effects. The critical size on the electronic effect of Pt was verified to be about 8 nm for this probe reaction. The above results were considered to reshape the research on the electronic and geometric effects of heterogeneous catalysis.

## Results and discussion

### Synthesis and characterization of Pt-based catalysts

**Fig. 1:**
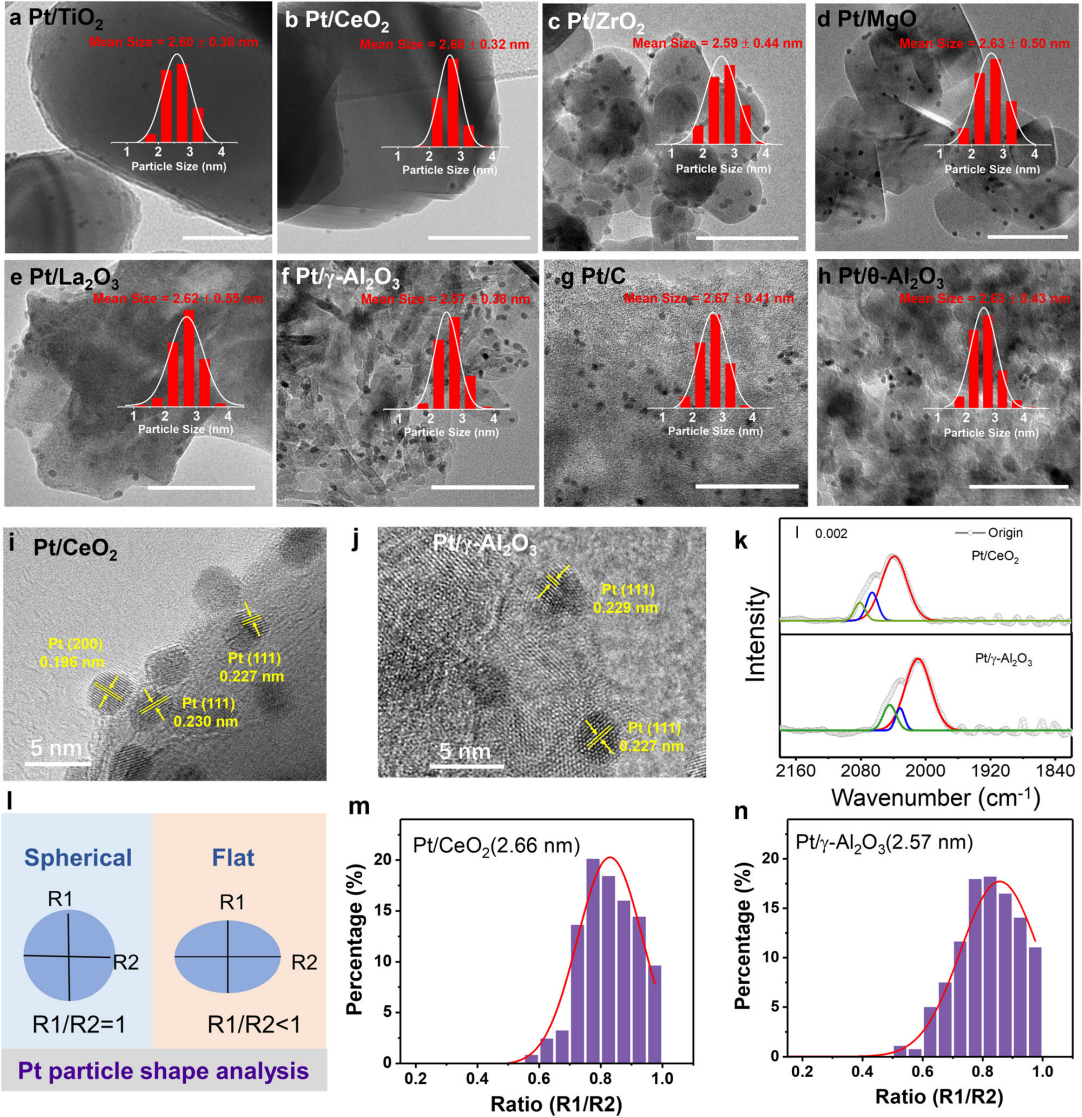
Morphology characterization of Pt-based catalysts. **a**–**h** HRTEM images of the size-controlled Pt catalysts over different supports and the inset are their size distribution histograms; scale bar: 50 nm. Representative HRTEM images of as-synthesized catalysts: Pt/CeO_2_ (**i**), Pt/γ-Al_2_O_3_ (**j**), and its corresponding infrared spectroscopy of CO adsorption (**k**). **l** Schematic presentation of the particle shape analysis based on the TEM images. The frequencies of Pt NPs diameter ratio distributions R1/R2 over CeO_2_ (**m**) and γ-Al_2_O_3_ (**n**).

PVP capped Pt NPs with controlled size of ~2.6 nm were synthesized by the modified colloidal method according to the literature^30^. The average size of the Pt NPs was approximately estimated as 2.6 nm with a narrow range seen from transmission electron microscope (TEM) images (Supplementary Fig. 1). After depositing these size-controlled particles on various supports, PVP was removed smoothly by facile NaBH_4_/tert-butylamine (TBA) treatment (Supplementary Fig. 2)^31^, as confirmed by FT-IR spectra (Supplementary Fig. 3)^28^. As seen in the high-resolution TEM (HRTEM) images and corresponding size distributions of Pt NPs (Fig. 1a–h), negligible difference in size compared to the unsupported Pt NPs indicated the successfully loading of the Pt NPs without aggregation or destruction. The lattice spacing of Pt NPs on representative supports as CeO_2_ and γ-Al_2_O_3_ was measured to be 0.23 nm, corresponding to the (111) plane of Pt crystal (Fig. 1i, j). The absence of diffraction peaks of metallic Pt in XRD patterns (Supplementary Fig. 4) also confirmed the high dispersion and uniformity of Pt NPs. The infrared spectroscopy of adsorbed CO was conducted to explore whether the shape of Pt NPs had changed considering the different metal-support interaction, selecting CeO_2_ and γ-Al_2_O_3_ as typical representatives of reducible and irreducible supports, respectively. As shown in Fig. 1k, although the peak position varied, similar absorption profiles for Pt/CeO_2_ and Pt/γ-Al_2_O_3_ were found. Both the patterns could be deconvoluted into three absorption peaks, ascribed to high- and low-coordinated sites on Pt NPs^32–34^. The calculated distribution of these sites with various coordination environments on the two samples using least square Gaussian-Lorentzian fit was also semblable, suggesting no obvious shape changes for Pt NPs after loading. Note that the blue shift of vibration frequencies for adsorbed CO on Pt/CeO_2_ compared with those of Pt/γ-Al_2_O_3_ indicates more electron-deficient property of Pt on CeO_2_^35,36^. Additionally, the shape of the Pt NPs was also analyzed following an approach described earlier to confirm the unchanged Pt shape^37,38^, which is based on the ratio of the shortest diameter (R1) to the longest diameter (R2) of two-dimensional projection of each Pt NP (Fig. 1l). The variation of R1/R2 ratios is expected to be related with the morphology change of particles. We evaluated more than 300 NPs for PVP stabilized Pt NPs, Pt/CeO_2_, and Pt/γ-Al_2_O_3_ based on the HRTEM images, respectively. These three samples exhibited a similar distribution of R1/R2 and the averaged R1/R2 value of the three samples were all fitted to be 0.83 (Fig. 1m, n and Supplementary Fig. 1), suggesting that the shape of Pt NPs was kept very well after loading on the supports. Besides the three samples, the ratios of R1/R2 were measured for the other catalysts. Similar averaged R1/R2 values supplied the unambiguous evidence on the undeformed Pt NPs (Supplementary Fig. 5).

**Fig. 2:**
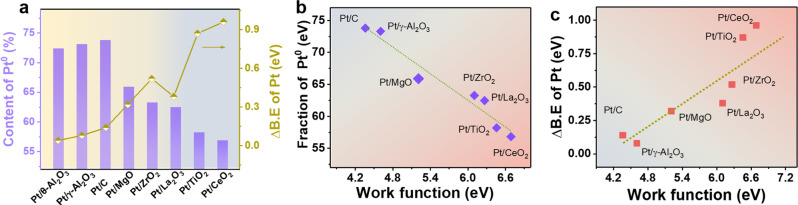
Electronic properties of platinum-based catalysts. **a** The fraction of Pt^0^ and the relative deviation of the standard binding energy of Pt 4*d* (or 4*f*) on different supported catalysts. Plot of the Pt^0^ content (**b**) and Δ*B*.*E*_Pt_ (**c**) for ~2.6 nm Pt-based catalysts against the *W*_f_ value of various supports, respectively.

According to previous works, the electronic structure of metal NPs would vary when changing the support material due to the altered charge transfer between metal and supports^39,40^. Then, X-ray photoelectron spectroscopy (XPS) were performed to investigate the support effect on the chemical states of Pt NPs (Supplementary Fig. 6) more comprehensively. In each XPS, peaks derived from Pt^0^ and Pt^2+^ were observed. The content of Pt^0^ and Pt^2+^ was estimated based on the area ratio of the Pt 4*d*_5/2_ peaks (4 *f*_7/2_ for MgO and ZrO_2_) (Fig. 2a). For irreducible supports including γ-Al_2_O_3_, θ-Al_2_O_3_, and activated carbon, more than 72% of the Pt species are present as Pt^0^, suggesting the slight charge transfer from Pt NPs to the support. When Pt NPs were loaded on reducible supports, however, the percentage of Pt^0^ species became obviously lower, with the fraction of Pt^0^ on Pt/TiO_2_ and Pt/CeO_2_ being just 57.7 and 56.8%, respectively, which meant that more electrons transferred from Pt to these two reducible supports. Pt NPs carried on other reducible supports displayed similar trends as those on TiO_2_ and CeO_2_. Δ*B*.*E*_Pt_, the relative deviation of the standard binding energy of Pt 4*d* (or 4*f*), was then proposed to further illustrate the oxidization state of supported Pt NPs. Easily apprehending that a positive value indicate the oxidative Pt. The higher the value, the more electrons transferring from Pt NPs to supports. As indicated in the Fig. 2a, the Δ*B*.*E*_Pt_ of Pt/CeO_2_ (0.96 eV) is the largest among all the catalysts, 0.88 eV higher than that of Pt/γ-Al_2_O_3_ (0.08 eV). And the variation of O 1 s core levels between supported Pt catalysts and the bare supports also supported the above results. (Detailed discussion can be seen in Supplementary Figs. 7–8 and Supplementary Note 2). The quasi in situ XPS was also employed here to investigate the chemical state of Pt NPs under the reaction condition (Supplementary Fig. 9). As indicated in the Supplementary Table 3, it can been seen that there were negligible difference on Δ*B*.*E*_Pt_ and the fraction of Pt^0^ between the ex-situ and quasi in situ XPS data, suggesting that the electronic structure of Pt NPs would not be effected by the mild reaction condition.

In order to extract further information of the support effect on the electronic structure of Pt NPs, representative Pt_19_ models with different supports were then built to simulate the charge transfer of Pt NPs by DFT calculation (Supplementary Fig. 10). As elucidated in Supplementary Fig. 11, Pt NPs carried on the reducible supports, such as CeO_2_ and TiO_2_, transfer more electrons to the supports, turning into more oxidative states when compared with those loaded on the irreducible ones. Among them, Pt NPs loaded on CeO_2_ transfer most electrons (0.74 e) to the supports. On the contrary, Pt NPs on the γ-Al_2_O_3_ and C, the representative irreducible supports, seem not to encounter obvious electron loss. However, it shoud be noted that the electrons transferred from Pt to ZrO_2_ were larger than those on other irreducible supports (γ-Al_2_O_3_ and C). And this is related to the easier reducibility of zirconia in the presence of a metal oxide interface^41^, which would promote electron transfer process. And the good linear correlation between the number of electron loss from Pt NPs and the variation of binding energy for O 1*s* indicated that the calculation results was reliable (Supplementary Fig. 12). Based on the above discussion, it is obvious that the electronic structure of Pt NPs would be altered by support materials and they are closely related.

Considering the fact that the size and shape of Pt nanoparticle almost unchanged, the difference in charge transfer between Pt and various supports should not originate from intrinsic factors of Pt itself but be caused by an extrinsic factor such as supports. One possible reason for this may be the occurrence of EMSI, in which the electrons between Pt NPs and supports can give rise to a rearrangement^9,42^. And the magnitude and direction of the charge transfer are driven by the differences between the Fermi level of metal NPs and support, ultimately seeking equilibrium of the electron chemical potentials^42^. Thus, the distinctions on the energy gaps of Fermi levels between NPs and supports were essential affecting EMSI process. Namely, the work function (*W*_f_) of support should be the intrinsic impactor regulating the charge transfer process behind the support effect since a larger *W*_*f*_ means a low Fermi level^43^. Then *W*_f_ of Pt_19_ cluster and all the supports were calculated (Supplementary Fig. 13). As expected, the *W*_f_ varies greatly (Supplementary Fig. 14). Much lower *W*_f_ were observed on reducible supports compared with those on irreducible ones. Taking the Fermi level of Pt_19_ cluster (−4.17 eV) as reference, relatively larger energy gaps of the Fermi levels were obtained for granted between Pt_19_ cluster and reducible supports compared with irreducible ones. In order to observe the effect of *W*_f_ on Pt electronic state more intuitively, *W*_f_ against the fraction of Pt^0^ and Δ*B*.*E*_Pt_ were plotted in Fig. 2b, c, respectively. It could be seen that the fraction of Pt^0^ decreased along with *W*_f_ of the support, while the Δ*B*.*E*_Pt_ would undergo a remarkably enhancement when *W*_f_ increased, suggesting that large energy gaps of the Fermi levels between NPs and reducible supports can aggrandize the electron transfer. It needs to be emphasized that this electronic interaction between metal and support was rather complicated and the supported metal particles could have a great influence on the reducibility of oxide support in some cases^41^, leading to the deviation of experimental data. Even so, the strong correlation between *W*_f_ and the fraction of Pt^0^ or Δ*B*.*E*_Pt_ well suggested that the *W*_f_ of support was a vital factor to determine the electron transfer and it is reliable to utilize it to describe the EMSI and further forecast the electronic state of Pt or the catalytic performance of the Pt-based catalysts according to the results illustrated in Fig. 2.

### Catalytic performance of Pt-based catalysts with various Pt size

**Fig. 3:**
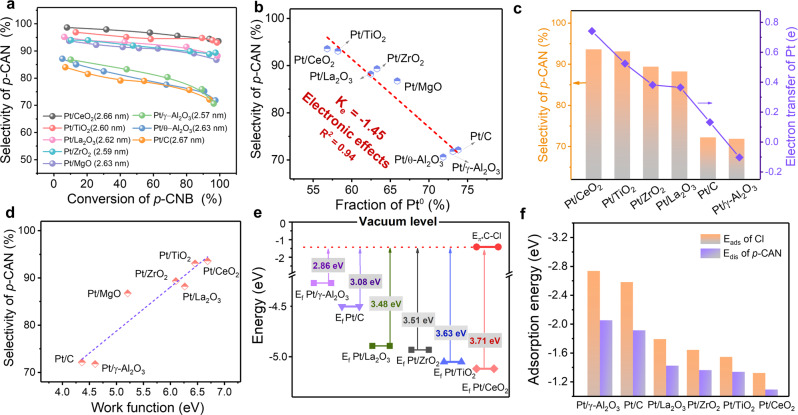
Correlating the electronic structure of Pt-based catalysts with the catalytic performance in chemoselective hydrogenation of *p*-CNB. **a** The selectivity to *p*-CAN as a function of *p*-CNB conversion over various catalysts with size-controlled Pt NPs in the size range of 2.6 nm. Reaction conditions: 10 mL toluene, 1 MPa H_2_, 45 °C, 0.5 mmol *p*-CNB, Pt/*p*-CNB: 10 wt‰; **b** plot of selectivity for *p*-CAN against the fraction of Pt^0^ on different supported Pt catalysts with the similar size range of ~2.6 nm. **c** Relationship between the selectivity of *p*-CAN and charge transfer of Pt NPs for the represent Pt models obtained by DFT calculation. **d** Plot of selectivity for *p*-CAN against the W_f_ of various supports. **e** The energy gap between the π* orbital of C–Cl bond and the Fermi levels of the representative Pt_19_-based models, respectively. The values represent Fermi levels against the vacuum. **f** The dissociation energies (*E*_dis_) of the *p*-CAN and the adsorption (*E*_ads_) energies of Cl on the Pt_19_-based catalyst models.

The hydrogenation of *p*-CNB was then exploited to evaluate the catalytic performance of the Pt-based catalysts supported on different supports (Supplementary Fig. 15). A moderate ratio of 10‰ for Pt/*p*-CNB was adopted here to get reliable results based on our previous report^25^. Obvious distinctions in the catalytic performance were observed on different catalysts under the same reaction condition (Fig. 3a). The selectivity to *p*-CAN can maintain over 90% at high conversions of *p*-CNB when Pt NPs were loaded over reducible supports. Hydrodehalogenation was almost entirely suppressed over Pt/CeO_2_ and Pt/TiO_2_. On the contrary, aniline (AN) was detected at the beginning of reaction and rose along with the reaction process on Pt/C, Pt/θ-Al_2_O_3_, and Pt/γ-Al_2_O_3_ (Supplementary Fig. 16). For instance, the selectivity of *p*-CAN on Pt/γ-Al_2_O_3_ was only about 69% when conversion reached 100%. The result clearly suggested that the selectivity of *p*-CAN was highly associated with supports, in which desirable *p*-CAN selectivity can be achieved over Pt NPs on reducible supports.

Considering the fact that several characterizations in this work have excluded the shape change of Pt NPs after loading, thus, the difference in selectivity should be ascribed to the electronic modification of Pt NPs from supports. Actually, the possibility to improve the selectivity of *p*-CAN by modulating the oxidation state of Pt NPs has been observed before^18,20^. The relationship between the selectivity of *p*-CAN and the fraction of Pt^0^ was then directly drawn in Fig. 3b. In general, as the fraction of Pt^0^ decreased, the selectivity of *p*-CAN increased reversely. A robust linear scaling relationship was found with a slope (*K*_e_) and *R*^2^ of −1.45 and 0.94, respectively. Notably, the slope (*K*_e-IN_) and *R*^2^_IN_ (−1.44 and 0.95 respectively) obtained based on the quasi in situ XPS data were quite similar to the ex situ ones (Supplementary Fig. 17). This linear relationship represents the electronic effect from supports without the involvement of geometric effect. To further examine the relationship between the selectivity of *p*-CAN and the oxidization state of Pt, *p*-CAN selectivity was then plotted against Δ*B*.*E*_Pt_ and the variation of the O 1*s*, respectively. Similar trends were also obtained as seen in Supplementary Figs. 18, 19. The analysis of Bader charge provided further evidence about this (Fig. 3c, Supplementary Fig. 20, and Supplementary Note 2), more electron-deficient Pt NPs showed higher selectivity. Since the electronic state of Pt has been proved to be closely associated with *W*_f_ of support earlier, the selectivity of *p*-CAN on Pt NPs of ~2.6 nm could also be correlated with the W_f_ of supports. As seen in Fig. 3d, the support with a higher *W*_f_ exhibited superior selectivity. The fair linear relationship between the selectivity and the W_f_ of support indicated that supports could directly manipulate the reaction performance through tuning the electronic structure of Pt by charge transfer.

Experimental observations discussed above had clearly demonstrated that the selectivity of the *p*-CAN was intimately related to the Pt oxidization state, which suggested that the cleavage of the carbon-halogen bonds was highly associated with the electronic structure of Pt NPs. Based on the Molecular Orbital Theory, it is necessary to fill electrons into the C–Cl π* orbitals to activate and further dissociate the C–Cl bond^44,45^. Therefore, the energy gap between the π* orbitals of the C–Cl bond and the Fermi energy of catalysts (*E*_f_) would be vital for the cleavage of the C–Cl bond. As shown in Fig. 3e, the π* orbital energy of the C–Cl bond was calculated to be −1.41 eV, higher than the *E*_f_ of all the catalysts. Pt catalysts with reducible supports demonstrated much lower *E*_f_ (<−4.88 eV) than those with irreducible counterparts (>−4.49 eV). The larger energy gap with the *E*_π*_ of C–Cl prevented the injection of *d* electrons from Pt NPs into the π* orbital of the C–Cl bond for reducible supports carried Pt NPs with relatively higher oxidation state. The dissociation energies (*E*_dis_) for the chemisorption of *p*-CAN as well as the adsorption energies (*E*_ads_) of a single chlorine atom on these models further confirmed the above inference (Fig. 3f and Supplementary Figs. 21–27).

The cleavage of C–Cl bond has been confirmed to be siginificantly affected by the electronic effect of Pt-based on the above statements and the metallic properties of Pt are more conducive to the activation and fracture of C-Cl bonds. According to previous work, large Pt NPs displayed more metallic properties since the charge transfer from Pt to support was sensitive to the size of Pt NPs^16^, which indicated that electron-rich Pt NPs of large size are more capable to transfer electrons to the π* orbital of the C–Cl bond and dissociate it sequentially. However, the cleavage of the C-Cl bond was also size-sensitive and would be suppressed on large Pt NPs due to the geometrical effect^24–26^. According to the literature, lack of compatible *d* valence orbitals over large Pt NPs make it impossible to match with the π* orbital of the C–Cl bond of *p*-CAN, and thus the cleavage process is inhibited^25^. Hence, a critical size should exist to switch the predominant factor governing the cleavage of the C–Cl bond from electronic effect to geometric effect.

**Fig. 4:**
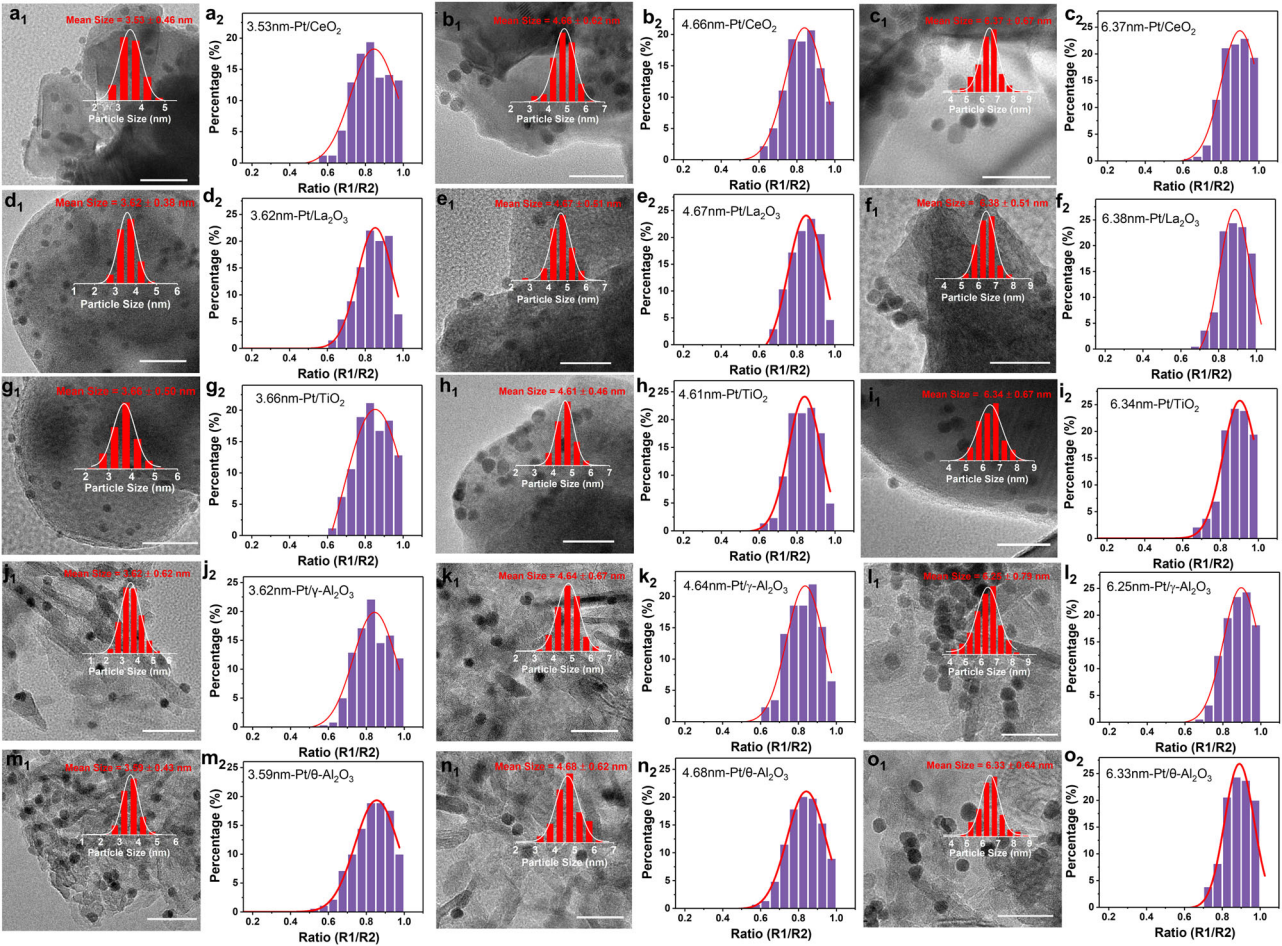
Structure characterization of Pt-based catalysts with varied size. Representative HRTEM images (**a**_**1**_–**o**_**1**_) of as-synthesized Pt/CeO_2_ (**a**–**c**), Pt/La_2_O_3_ (**d**–**f**), Pt/TiO_2_ (**g**–**i**), Pt/γ-Al_2_O_3_ (**j**–**l**), and Pt/θ-Al_2_O_3_ (**m**–**o**) catalysts with varied Pt particle sizes and its corresponding ratio of R1/R2 (**a**_**2**_–**o**_**2**_). **a** 3.53 nm-Pt/CeO_2_. **b** 4.66 nm-Pt/CeO_2_. **c** 6.37 nm-Pt/CeO_2_. **d** 3.62 nm**-**Pt/La_2_O_3_. **e** 4.67 nm-Pt/La_2_O_3_. **f** 6.38 nm-Pt/La_2_O_3_. **g** 3.66 nm-Pt/TiO_2_. **h** 4.61 nm-Pt/TiO_2_. **i** 6.34 nm-Pt/TiO_2_. **j** 3.62 nm-Pt/γ-Al_2_O_3_. **k** 4.64 nm-Pt/γ-Al_2_O_3_. **l** 6.25 nm-Pt/γ-Al_2_O_3_. **m** 3.59 nm-Pt/θ-Al_2_O_3_. **n** 4.68 nm-Pt/θ-Al_2_O_3_. **o** 6.33 nm-Pt/θ-Al_2_O_3_. Insets in the (**a**_**1**_–**o**_**1**_) were the corresponding size distributions. The scale bar in all the figures are 20 nm.

Thus, Pt NPs with larger sizes (~3.6, ~4.6, and ~6.3 nm) were synthesized and further loaded over CeO_2_, La_2_O_3_, TiO_2_, γ–Al_2_O_3_, and θ-Al_2_O_3_ (Supplementary Figs. 28–37). Similar with the ~2.6 nm Pt catalysts, both the HRTEM images and R1/R2 analysis of these larger Pt NPs indicated the negligible shape change of Pt NPs when loaded on different supports (Fig. 4). XPS data showed that the fraction of Pt in high valence states over all these supports fell off along with the rise of the size of Pt NPs, suggesting that charge transfer is attenuated for larger Pt NPs^16^ (Supplementary Figs. 35–37). As a result, the fraction of Pt^0^ rose remarkably from 56.8% and 73.1% to 82.4% and 90.5%, correspondingly, as the size of Pt NPs increased from 2.6 to 6.3 nm over CeO_2_ and γ-Al_2_O_3_, respectively (Supplementary Fig. 35c). Meanwhile, the fraction of Pt^0^ on irreducible supports remained higher than those on reducible supports with the same particle size, consistent with the situation of ~2.6 nm NPs above.

**Fig. 5:**
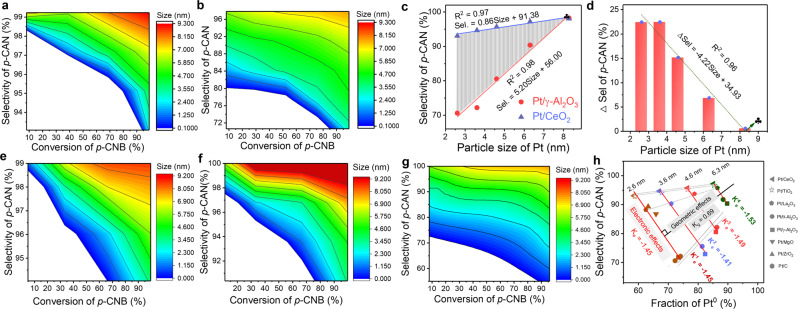
Decoupling the electronic and geometric effects of Pt catalysts in chemoselective hydrogenation of *p*-CNB. The selectivity to *p*-CAN as a function of conversion against various parictie sizes of Pt over Pt/CeO_2_ (**a**), Pt/γ-Al_2_O_3_ (**b**), Pt/TiO_2_ (**e**), Pt/La_2_O_3_ (**f**) and Pt/θ-Al_2_O_3_ (**g**). **c** Selectivity of *p*-CAN at nearly full converison of *p*-CNB over Pt/CeO_2_ and Pt/γ-Al_2_O_3_ with various parictie sizes of Pt. **d** The ∆Sel of *p*-CAN between Pt/CeO_2_ and Pt/γ-Al_2_O_3_ with similar particle size. **h** The selectivity of *p*-CAN against the fraction of Pt^0^ over different catalyst with various parictie sizes of Pt. The special marks in figures (**c**) and (**d**) represents the theoretical critical size of Pt. Reaction conditions: 10 mL toluene, 1 MPa H_2_, 45 °C, 0.5 mmol *p*-CNB, Pt/*p*-CNB: 10 wt‰.

The catalytic performance of Pt NPs with various sizes mentioned above were then evaluated under identical reaction conditions in *p*-CNB hydrogenation reaction (Fig. 5 and Supplementary Figs. 38, 39). In line with our pervious report^25^, Pt NPs over CeO_2_ delivered more desirable selectivity towards *p*-CAN than that on γ-Al_2_O_3_. Meanwhile the selectivity of *p*-CAN increased linearly with the rise of Pt particle size both on CeO_2_ and γ-Al_2_O_3_ (Fig. 5c). As the size of Pt NPs increased from 2.66 to 6.37 nm over CeO_2_, the selectivity increased from 93.1 to 97.2%. And it increased from 70.6 to 90.3% for Pt NPs on γ-Al_2_O_3_ when the size increased from 2.57 to 6.25 nm. An obvious distinction in selectivity between Pt/CeO_2_ and Pt/γ-Al_2_O_3_ can be found over the same size of Pt NPs, and the selectivity discrepancy (∆Sel) between Pt/CeO_2_ and Pt/γ-Al_2_O_3_ decreased rapidly as Pt NPs grew up (Fig. 5d). When the particle size of Pt reached ~6.3 nm, ∆Sel reduced to only 6.9%. Since the geometry of Pt NPs has been proved to be changed little at each size, ∆Sel reflects the influence of electronic structure of Pt on *p*-CAN selectivity over different catalysts, which can also be called the electronic effect of the catalyst. In other words, the shadow region in Fig. 5c represents the pure relative electronic effects of catalysts under various sizes of Pt. Apparently, the electronic effects of catalyst faded gradually when rising the particle size. And the critical value of Pt particle size was determined to be about 8 nm for *p*-CNB hydrogenation on Pt catalysts when extending the fitted linear curves, where the geometric effects became dominant alone. In order to verify this critical size of Pt, 8.21 nm-Pt/CeO_2_ and 8.28 nm-Pt/γ-Al_2_O_3_ were then prepared (Supplementary Figs. 40–43). These two catalysts show a negligible difference in selectivity of *p*-CAN under the same conditions with ∆Sel of *p*-CAN less than 1%, even though the content of Pt^0^ is different between the two catalysts (Supplementary Fig. 35). This result manifested that *p*-CAN selectivity was almost independent of supports when the particle size of Pt was larger than 8.2 nm. In other words, the electronic effect would not cause remarkable effects on the *p*-CAN selectivity, while the geometric effects of Pt became dominant alone over this critical size. The desirable selectivity of p-CAN (>99.9%) obtained over 36 nm-Pt/CeO_2_ and 36 nm-Pt/γ-Al_2_O_3_ further validated the above conclusion (Supplementary Figs. 44–46).

### Decoupling the electronic and geometric effects of Pt catalysts

Perhaps the biggest challenge is to decouple the electronic and geometric effects below the critical size of Pt NPs. More specifically, it is still not clear about the changing pattern of the electronic effect with the particle size. To solve this issue, the catalytic data of Pt/CeO_2_, Pt/γ-Al_2_O_3_, Pt/TiO_2_, Pt/La_2_O_3_, and Pt/θ-Al_2_O_3_ with particle sizes ranging from 2.6 to 6.3 nm were then plotted with the fraction of Pt^0^. This is the critical procedure in this work. As seen in Fig. 5h, the linear relationships were also found for these 5 kinds of catalysts of various Pt particle sizes, which is usually called the size effect curves. What surprised us is that the slopes of the electronic effect at different sizes of Pt NPs were rather similar. Namely, the linear relationships between the selectivity of *p*-CAN and Pt^0^ at different sizes of Pt NPs were parallel with each other. This phenomenon indicated that the state function of the electronic effect is independent with the particle size of Pt of controlled shapes and clearly determined by the bond nature of C–Cl itself in *p*-CAN catalyzed by Pt. Since *K*_e_ of the state function of the electronic effect has been given to be −1.45 earlier in Fig. 3b, the slop of the state function of geometric effect was then determined to be 0.69 (*K*_g_) according to the orthogonal relation from a mathematical or physical point of view. Coming to this step, the electronic and geometric effects were then quantitatively decoupled for the first time. When switching to the new set of rectangular axes composed of the state functions of the electronic and geometric effects, the specific relative values of these two effects can then be obtained by projecting the traditional fitted size effect lines for Pt catalysts of specific supports to the new axes.

One can find that the electronic effect shown in Fig. 3b only reflects the external electronic modulation to Pt NPs such as the support induced charge transfer. According to the Wulff construction rule^11^, the ratio of low coordinated sites (e.g., edge and corner sites) and high coordinated ones (terrace sites) would change a lot along with the particle size and shape, which can also greatly affect the catalytic performance. This is usually called the geometric effect. However, this effect is always accompanied by the change of the energy bands in metal NPs and charge transfer between metal NPs and supports when the size of metal NPs varies, which is hardly to be split off. Hence the size and shape induced parts of electronic effect was grouped together with the geometric effect in this work. When coming to the concrete research process, the shape and size of Pt NPs is strictly controlled to be similar since single variable rule has to be obeyed during the constructing process of a new set of rectangular axes. If not, the data points that make up the linear relationship representing the electronic effect would be rather discrete. Then the orthogonal relationship can never be found. Once the new rectangular axe is built, the two parts of the geometric effect is naturally included. For example, when the shape of metal NPs for a given size is changed, its corresponding behavior is expected to deviate from the state function of the electronic effect.

To sum up, the electronic and geometric effects were decoupled for the first time in *p*-chloronitrobenzene hydrogenation probe reaction on size- and shape-controlled Pt catalysts. A linear relationship between the selectivity of *p*-CAN and *W*_f_ of support was found. Supports with large W_f_ can effectively attenuate the ability of Pt NPs to fill valence electrons into the π* orbital of the C–Cl bond and passivate the hydrogenolysis side reaction. A series of linear scaling relationships reflecting the electronic effect was also found on various sizes of Pt NPs on different supports. Through the orthogonal decomposition method, the geometric effect was successfully spun off based on a series experimental data. Afterwards separable nested double coordinate system was developed to quantitatively evaluate the two effects. In addition, the critical size determining the dominant role between the two effects was verified to be ~8 nm for this reaction. To be prospective, future studies using in-situ characterization techniques, such as XANES, are greatly encouraged to further quantify the fraction of Pt^0^ or the electronic structure of Pt under reaction conditions.

## Methods

### Chemical and materials

γ-Al_2_O_3_ was obtained by thermal decomposition of boehmite (Sasol, PURAL alumina) at 500 °C for 1 h. θ-Al_2_O_3_, ZrO_2_, CeO_2_, TiO_2_, and MgO were calcined at 500 °C for 1 h. La_2_O_3_ were calcined at 600 °C for 1 h. Those oxide supports were all purchased from rom Aladdin Inc. The active carbon was purchased from Jiangsu Zhuxi Activated Carbon Co., LTD. And their BET surface area were showed in Supplementary Table 1. H_2_PtCl_6_•6H_2_O (Pt ≥ 37.5 wt%), tert-butylamine (TBA, 99.5%), *p*-chloronitrobenzene (*p*-CNB), *p*-chloroaniline (*p*-CAN), N,N-dimethylformamide (DMF), acetone and 2-propanol were all purchased from Aladdin Inc.

### Synthesis of Pt nanoparticles

The Pt nanoparticles with the average size of 2.6 and 3.6 nm were synthesized by modified colloidal methods previously reported by Cargnello et al.^30^. In a typical procedure, 17 mg PVP (*M*_w_ = 58000) was dissolved in 45 mL propanol, and then 5.0 mL H_2_PtCl_6_ aqueous solution (6.0 mM) was added drop by drop. After stirring for about 2 min at room temperature, the solution was refluxed in a 100 mL flask for 3 h under air at 90 °C to synthesize the PVP-stabilized Pt NPs. The as-synthesized Pt NPs can be used directly without further treatment. The synthesis of 3.6 nm Pt nanoparticles was using the similar method. H_2_PtCl_6_•6H_2_O and PVP were dissolved in ethylene glycol in a 1:4.4 ratio by mass. The solution was then heated to 165 °C for 1 h under an argon atmosphere. The acetone was then added into the resulting dark brown solution to precipitate the particles from solution. The obtained Pt particles were washed three times using acetone and subsequently dissolved in ethanol (Pt: 0.5 mg/ml). The preparation of Pt particles at 4.6 nm was similar to that mentioned above. And the ratio of H_2_PtCl_6_•H_2_O and PVP was changed to 1:4 by mass and the heat temperature increased to the 180 °C^46^. The platinum nanoparticles with the average size of 6.4 nm used here were synthesized using the modified seeded growth method^47^. First, 60 mg H_2_PtCl_6_•6H_2_O was dissolved in 50 mL of ethylene glycol (EG, Fluka, ≥99.5%) solution which containing 132 mg PVP (*M*_w_ = 58000). The mixture was placed in a 100 mL three-neck flask and heated in a preheated oil bath at 160 °C for 5 h under the Ar atmosphere, and further used as the Pt seed solution to synthesize the larger nanoparticles (6.4 nm). Typically, 10 ml H_2_O were added into 10 ml of the Pt seed solution to form the mixture A. Subsequently, 38.5 mL of a 6 mM H_2_PtCl_6_ solution was then added in to the mixture A over time (30 min) while H_2_ was passing through the reaction vessel at 100 °C. The H_2_ was bubbled for an additional 1 h after the addition of the platinum precursor. And then the final mixture heated with a temperature-controlled electric oven at 100 °C for another 24 h. The supernatant was separated and precipitated by adding a triple volume of acetone, followed by centrifugation at 10,000 rpm (10,974 × *g*) for 15 min. The precipitate was collected and redispersed in ethanol (Sigma-Aldrich, ≥99.5%) with sonication. The preparation of Pt particles with the size of 8.2 nm was similar to that of 6.4 nm. 10 mL of 35 mg/mL H_2_PtCl_6_ solution was then added to the mixture A over time (30 min) while H_2_ was passing through the reaction vessel at 100 °C. And then the final mixture heated with a temperature-controlled electric oven at 180 °C for another 24 h. Other operation was similar to that of 6.4 nm. The Pt NPs with an average size of 36 nm were prepared by adding 2 ml N_2_H_4_ into 1.6 ml 10 mg/mL H_2_PtCl_6_ solution. The solution was used without further treatment.

### Synthesis of PVP-Pt/support catalysts

In a typical procedure, a certain amount of as-synthesized Pt NP solution (the theoretical load of Pt was 0.5 wt%) was added drop by drop into 10 mL DMF of the support under vigorous stirring, and then the mixed solution was further stirred at room temperature for 4 h. Subsequently, PVP-Pt/support catalyst was collected by centrifugation at 5000 rpm (2743 × *g*) for 10 min and washed with acetone. The 36nm-Pt/CeO_2_ and 36nm-Pt/Al_2_O_3_ catalysts were prepared by impregnation method.

### PVP removal

The removal of capping agent PVP from the surface of PVP-Pt/support was carried out using a NaBH_4_/TBA aqueous solution according to the previous article^31^. In a typical process, 300 mg as-prepared PVP-Pt/support catalysts (the theoretical loading of Pt was 0.5 wt%) were treated in 6.2 mL NaBH_4_/TBA aqueous solution (NaBH_4_: TBA: H_2_O = 10 mg: 5 mL: 1.2 mL) with stirring for 1 h at low temperature (~275 K). These samples (denoted as Pt/support) were collected by centrifugation at 3000 rpm (987 × *g*) for 10 min after TBA treatment. The collected sample was then washed three times with acetone solution to remove the excess surface adsorbed amine. And after drying under 80 °C in the vacuum oven, the sample can be used as the hydrogenation catalysts.

### Characterization

TEM images were recorded on a Hitachi HT-7700 microscope. A JEOL JEM-2100F was used to characterize the HRTEM and HAADF-STEM. XPS was conducted on an Escalab 250Xi spectrometer and calibrated with C 1*s* (284.6 eV) to measure the chemical valence of all the elements. Quasi in-situ XPS measurement: Quasi in situ XPS measurements were conducted with a Thermo Scientific K-Alph^a+^ X-ray photoelectron spectrometer. The monochromatic Al K^a^ (1486.6 eV) X-ray source was used at 225 W (15 kV, 15 mA). The catalysts were pre-treated in the reduction conditions (45 °C, 1 MPa H_2_) and kept as long as the reaction time used in the hydrogenation reaction. Next, the sample was cooled in a glove box with Ar atmosphere, then collected and transferred to the analyzer chamber for XPS analysis without exposing to air. The inductively coupled plasma-atomic emission spectrometry (ICP-AES) was carried out on a Perkin Elmer Optima OES 800. The Powder X-ray diffraction (XRD) was performed using a wide-angle X-ray diffractometer (D/tex-Ultima TV) with Cu Kα radiation (λ = 1.54 Å) (tube operated at 40 kV and 40 mA). The Pt content in the prepared catalysts was analyzed by inductively coupled plasma–atomic emission spectroscopy (ICP-AES, PerkinElmer 3300 DV). The textual properties were collected on a Micromeritics ASAP 2020 HD88 instrument. The specific surface area was calculated by the Brunauer-Emmett-Teller (BET) method. Prior to analysis, the samples were degassed in vacuum at 573 K for 12 h. The contents of the liquid were analyzed by GC (FULI, GC-9750) and the products were identified by GC-MS (Agilent Technologies, GC 6890 N, MS5970). The in situ FTIR spectra for CO adsorption were recorded on a Vertex 70 equipped with an in situ heating cartridge. Before CO adsorption, the sample was pre-treated in Ar flow at 200 °C for 2 h, and then cooled to 30 °C for CO adsorption. CO adsorption was carried in the CO/He mixture (10% CO) with the flow 20 mL·min^−1^ for 2 h. Subsequently, the CO was switched to He with the flow rate of 20 mL·min^−1^ to sweep the sample. Spectra were collected every five minutes at 30 °C with a resolution of 4 cm^−1^ and accumulation of 32 scans.

### Catalytic reaction

The hydrogenation of *p*-CNB was carried out in a 50 mL stainless steel high-pressure batch reactor. Typically, *p*-CNB (0.5 mmol), the catalysts and toluene (10 mL) were loaded into the autoclave. The ratio of Pt/*p*-CNB was set as 10wt‰ in all tests. Firstly, the reactor was purged three times with pure H_2_ to remove air. After that, the reactor was charged with 1 MPa H_2_ and the reaction mixture was stirred at 45 °C. After the reaction, the reactor was placed into a water bath and cooled to room temperature. The remaining H_2_ was carefully vented and the reaction mixture was centrifuged to obtain the supernatant and the supernatant was followed to be analyzed by GC.

### Computational methods

Calculations were performed by using periodic, spin-polarized DFT as implemented in the Vienna ab initio program package (VASP)^48,49^, using the projector augmented wave (PAW) method proposed by Blöchl^50^ and implemented by Kresse^51^. A cutoff energy of 400 eV for plane waves was set through all the calculations and exchange correlation functional approximation was treated with PBE functional^52^. A Gaussian electron smearing method with σ = 0.05 eV were used. A p (3 × 4) supercell with a 4-layer slab of 144 atoms for CeO_2_ (110) were modeled while p (3 × 5) with eight-layer slabs of 180 atoms was used for TiO_2_ (101) model. The γ-Al_2_O_3_ supercell were built according the previous reporter^53^ and a p (2 × 3) supercell with a 7-layer slab of 240 atoms for γ-Al_2_O_3_(100) were used for substrates. p (6 × 5) supercell with a 3-layer slab of 180 atoms for ZrO_2_ (101) and p (5 × 5) supercell with a 4-layers slab of 200 atoms for MgO (100) were used for ZrO_2_ and MgO, respectively. The La_2_O_3_ were modeled using a p (5 × 5) supercell for La_2_O_3_ (001) with a 5-layer slab containing 125 atoms. A p (7 × 7) graphene supercell containing 98 atoms was considered as the hierarchical porous active carbon. A k-point of 2 × 2 ×1 was used for the Brillouin zone sampling for structure optimization. The periodic condition was employed along the *x* and *y* directions and the vacuum region between the slabs is 15 Å, which is sufficiently large to keep spurious interactions negligible. The upper two or four layer atoms for CeO_2_ (110)/La_2_O_3_ (001)/MgO (100) and TiO_2_ (101)/γ-Al_2_O_3_ (100)/ZrO_2_ (101), in the supercells are allowed to relax. Meanwhile, the bottom two or three (four layers for TiO_2_ (101)/ZrO_2_ (101)) layer atoms were fixed during the structure optimization. The geometry optimization was stopped when the force residue on the atom is smaller than 0.02 eV Å^−1^ and the energy difference was <10^−4^ eV. The work functions of supports were calculated according to the previous literature^54,55^. The adsorption and dissociation energies for molecule chemisorption are defined as follows respectively:

1 $${{E}}_{{{{{{\rm{ads}}}}}}}={{E}}_{{{{{{\rm{tota}}}}}}}-{{E}}_{{{{{{\rm{slab}}}}}}}-{{E}}_{{{{{{\rm{mol}}}}}}}$$

2 $${{E}}_{{{{{{\rm{dis}}}}}}}={{E}}_{{{{{{\rm{totd}}}}}}}-{{E}}_{{{{{{\rm{ads}}}}}}}$$

where *E*_tota_ (*E*_totd_) is the total energy after a molecule adsorption (dissociation) on the catalyst; *E*_slab_ is the energy of the clean catalyst alone; *E*_mol_ is the energy of the molecule in the gas phase.

The work function (*W*_f_) was calculated by:

3 $${{W}}_{{{{{{\rm{f}}}}}}}=\left|{{E}}_{{{{{{\rm{fermi}}}}}}}-{{E}}_{{{{{{\rm{vacuum}}}}}}}\right|$$

where *E*_vacuum_ is the energy level of the vacuum, and *E*_fermi_ denotes the Fermi energy level of the support or the catalyst.

## Supplementary information

Supplementary Information

## Data Availability

The data that support the findings of this study can be found in the manuscript and Supplementary Information, or are available from the corresponding authors upon request.
