# Supplementary material for: Decoupling the electronic and geometric effects of Pt catalysts in selective hydrogenation reaction
Source: Nat Commun. 2022 Jun 21;13:3561. doi: 10.1038/s41467-022-31313-4 (PMC9213482; doi:10.1038/s41467-022-31313-4)
Supplement: Supplementary file 1 — Supplementary Information [file 41467_2022_31313_MOESM1_ESM.pdf]

# Supplementary Information

## Decoupling the Electronic and Geometric Effects of Pt Catalysts in Selective Hydrogenation Reaction

Zhe Wang,[1,2,3], Chunpeng Wang[1,3], Shanjun Mao[1*], Bing Lu[1], Yuzhuo Chen[1], Xie Zhang[1], Zhirong Chen[2], Yong Wang[1*]

[1]Advanced Materials and Catalysis Group, Center of Chemistry for Frontier Technologies, State Key Laboratory of Clean Energy Utilization, Institute of Catalysis, Department of Chemistry, Zhejiang University, Hangzhou 310028, P. R. China.

[2]College of Chemical and Biological Engineering, Zhejiang University, Hangzhou 310028, P. R. China.

[3]These authors contributed equally: Zhe Wang, Chunpeng Wang.

*Corresponding authors E-mail: maoshanjun@zju.edu.cn; chemwy@zju.edu.cn

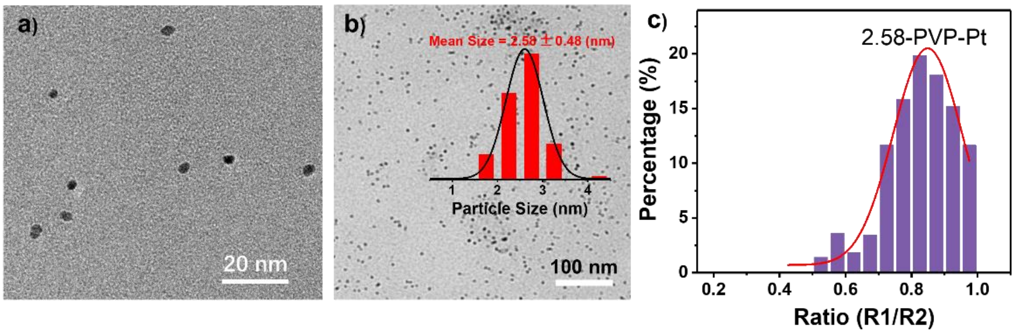


**Supplementary Fig. 1. TEM characterization of the PVP-capped Pt NPs.** (a, b) TEM images of the PVP-protected Pt particles and their size distribution histograms (inset in b). (c) The frequencies of Pt NPs diameter ratio distributions R1/R2 of the PVP-protected Pt NPs with the average size of 2.58 nm.

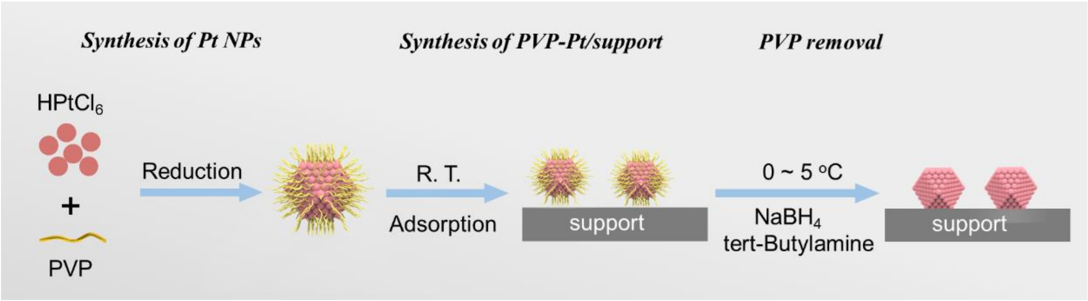


**Supplementary Fig. 2. Synthetic route of Pt-based catalysts.** Schematic illustration showing the procedure to prepare Pt-based catalysts.

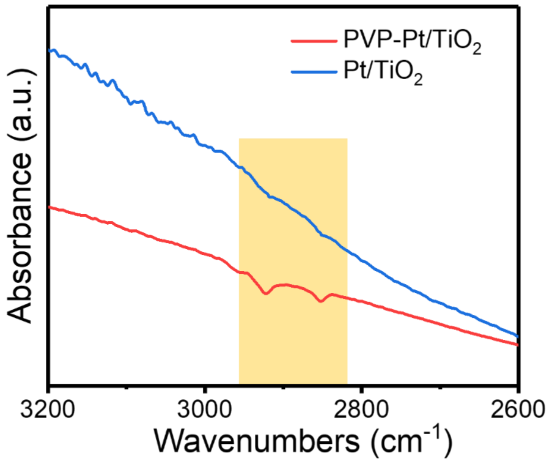


**Supplementary Fig. 3**. **FT-IR of different Pt/$TiO_2$ catalysts.** FT-IR spectrum of the Pt catalyst supported on $TiO_2$ catalyst before and after TBA treatment.

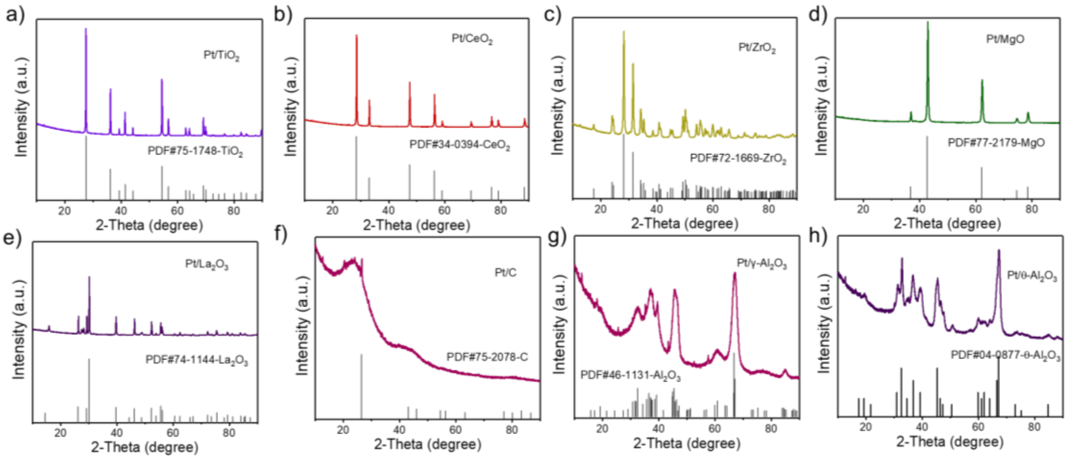


**Supplementary Fig. 4**. **XRD patterns of Pt-based catalysts**. XRD patterns of the various catalysts with the average Pt size of 2.6 nm. (a) Pt/$TiO_2$, (b) Pt/$CeO_2$, (c) Pt/$ZrO_2$, (d) Pt/MgO, (e) Pt/$La_2O_3$, (f) Pt/C, (g) Pt/γ-$Al_2O_3$, (h) Pt/θ-$Al_2O_3$.

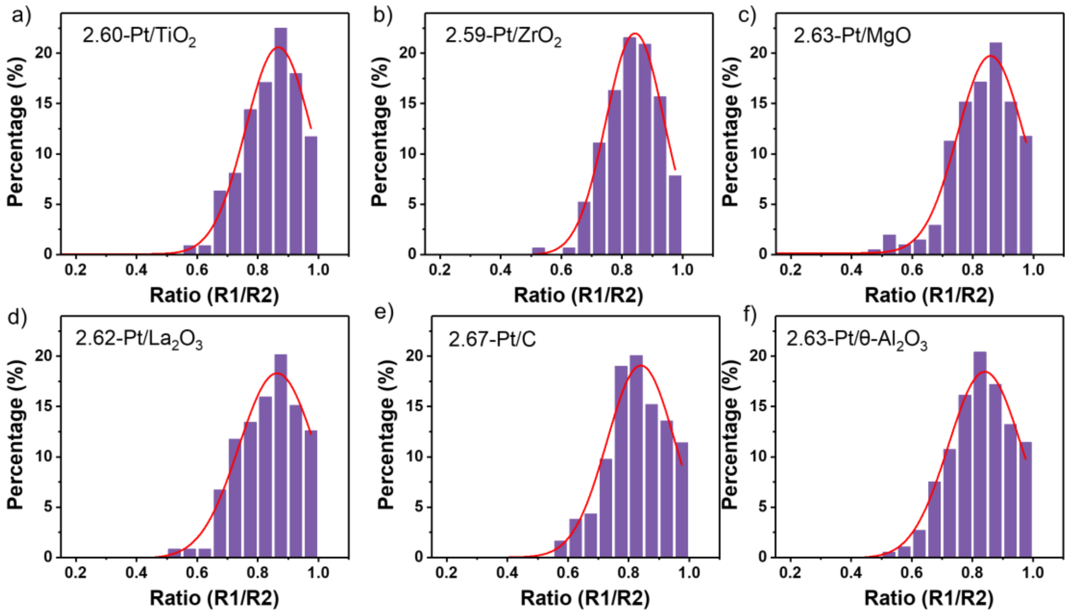


**Supplementary Fig. 5. Analysis of R1/R2 for the Pt NPs over different catalysts.** The frequencies of Pt NPs diameter ratio distributions R1/R2 over altered supported Pt catalysts with the average Pt size of 2.6 nm. (a) Pt/$TiO_2$, (b) Pt/$ZrO_2$, (c) Pt/MgO, (d) Pt/$La_2O_3$, (e) Pt/C, (f) Pt/θ-$Al_2O_3$.

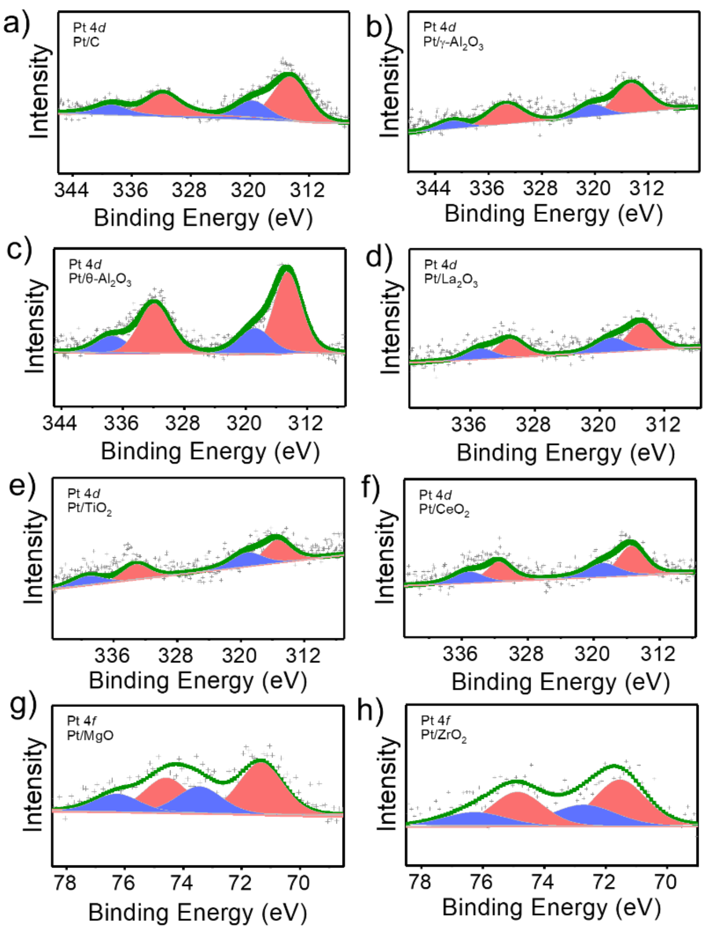


**Supplementary Fig. 6. XPS spectra of series Pt-based catalysts.** XPS spectra of Pt 4*d* (a-f) and 4*f* (g-h) in the different Pt catalysts with the Pt size of ~2.6 nm, respectively. (a) Pt/C, (b) Pt/γ-$Al_2O_3$, (c) Pt/θ-$Al_2O_3$, (d) Pt/$La_2O_3$, (e) Pt/$TiO_2$, (f) Pt/$CeO_2$, (g) Pt/MgO, (h) Pt/$ZrO_2$.

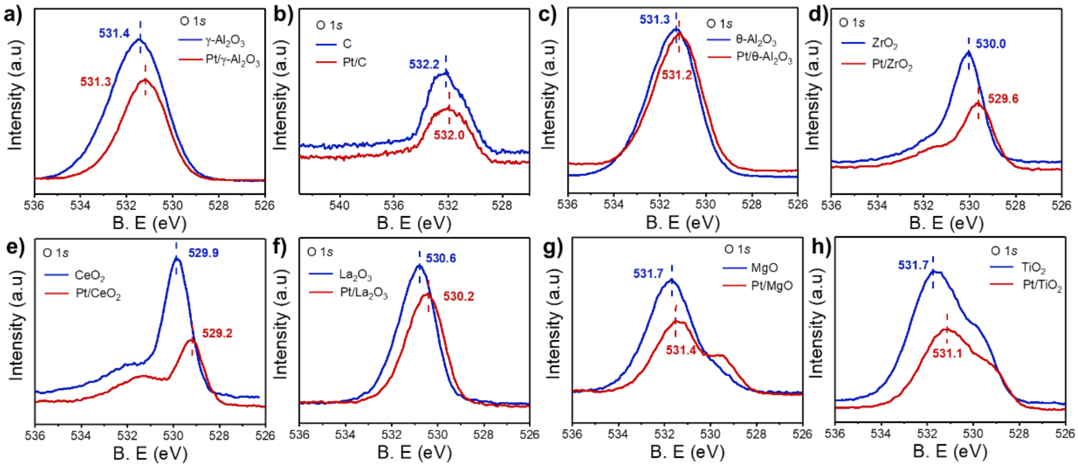


**Supplementary Fig. 7. XPS spectra of Pt-based catalysts and the corresponding supports.** XPS spectra of O 1*s* in the different carriers (blue) and Pt-supported catalysts (red), respectively. (a) Pt/γ-$Al_2O_3$, (b) Pt/C, (c) Pt/θ-$Al_2O_3$, (d) Pt/$ZrO_2$, (e) Pt/$CeO_2$, (f) Pt/$La_2O_3$, (g) Pt/MgO, (h) Pt/$TiO_2$.

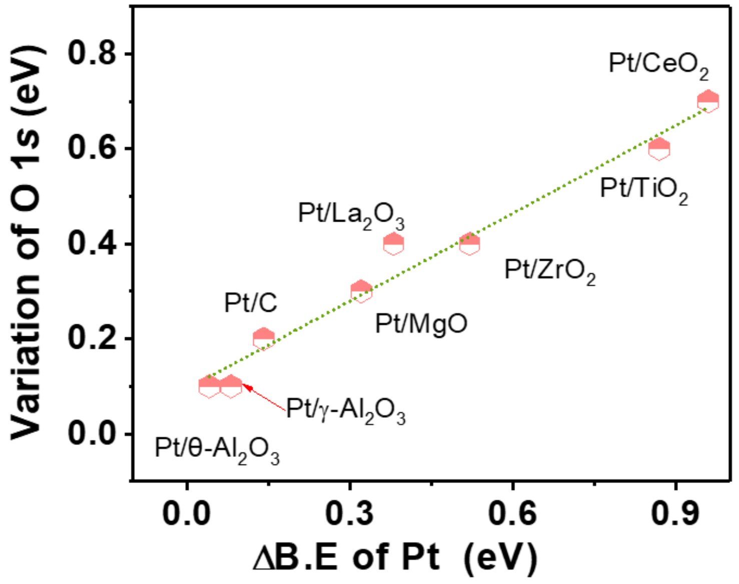


**Supplementary Fig. 8. Analysis of Pt and O XPS data.** The correlation between $\Delta B.E_{Pt}$ and variation of O 1*s* core level of various supports before and after Pt loading.

**Supplementary Note 1:** The variation of O 1*s* core levels between the supported Pt catalysts and the bare supports can also be an alternative explanation for describing the interaction between Pt NPs and diverse supports. The O 1*s* core level of various oxide supports were then acquired to provide the further evidence about the charge transfer (Supplementary Fig. 8). Comparing the O 1*s* binding energy before and after loading, it can be seen that the O 1*s* peak was shifted to a lower energy in all catalysts. However, what should be noted is that the variation of O 1*s* is more significant for the Pt catalysts carried on the reducible supports and displayed a good linear relationship with the $\Delta B.E_{Pt}$, which confirms again that more charge transfer occurred between Pt NPs and reducible supports in comparison to the irreducible ones.

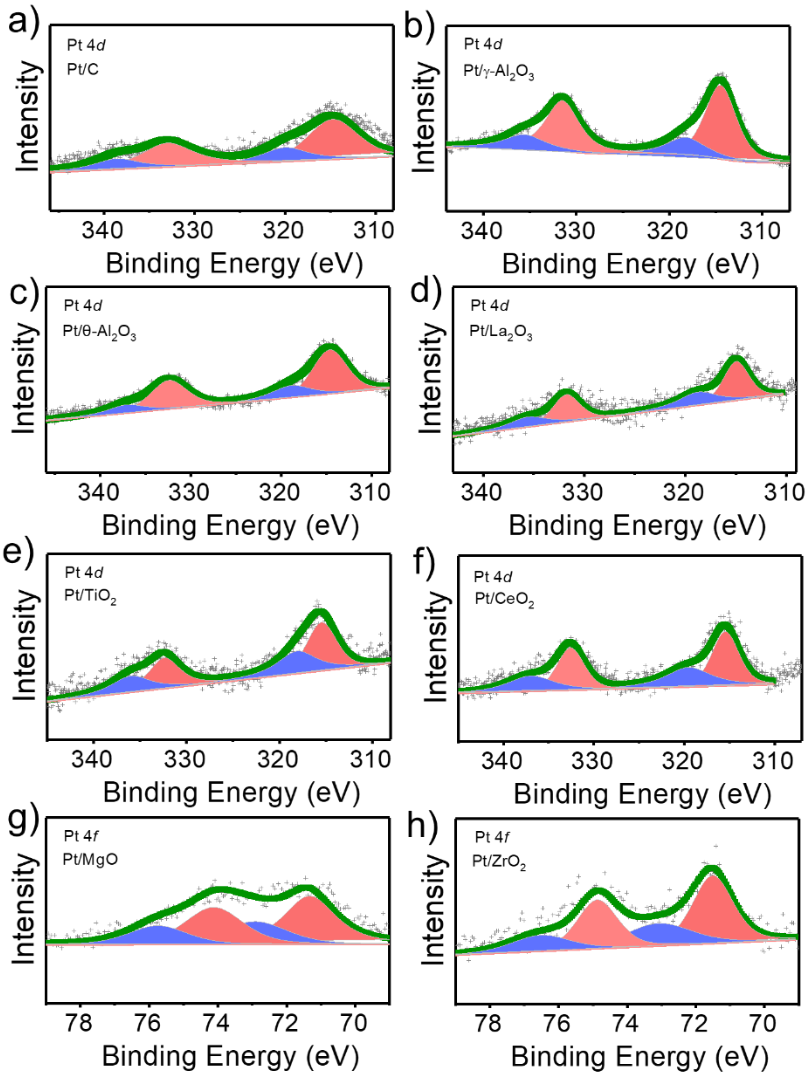


**Supplementary Fig. 9. Quasi *in-situ* XPS spectra of Pt-based catalysts.** Quasi *in-situ* XPS spectra of Pt 4*d* (a-f) and 4*f* (g-h) in the different Pt catalysts with the Pt size of ~2.6 nm, respectively. (a) Pt/C, (b) Pt/γ-$Al_2O_3$, (c) Pt/θ-$Al_2O_3$, (d) Pt/$La_2O_3$, (e) Pt/$TiO_2$, (f) Pt/$CeO_2$, (g) Pt/MgO, (h) Pt/$ZrO_2$.

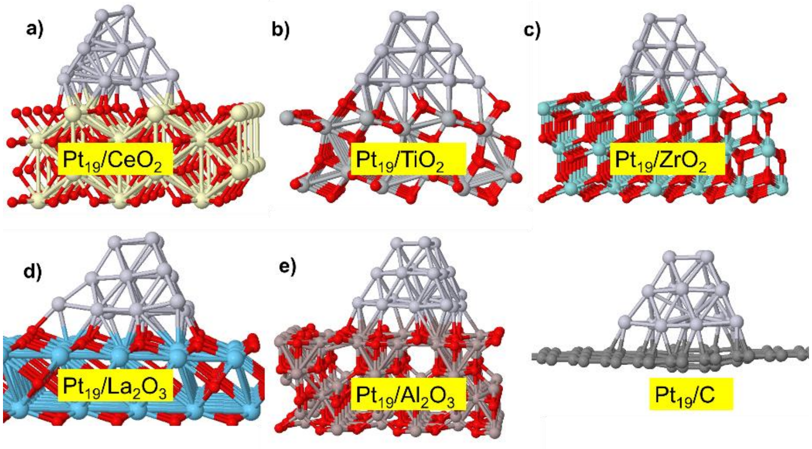


**Supplementary Fig. 10. DFT models of representative catalysts.** The optimized models of the representative Pt-based catalysts. (a) $Pt_{19}/CeO_2$, (b) $Pt_{19}/TiO_2$, (c) $Pt_{19}/ZrO_2$, (d) $Pt_{19}/La_2O_3$, (e) $Pt_{19}/Al_2O_3$, (f) Pt/C.

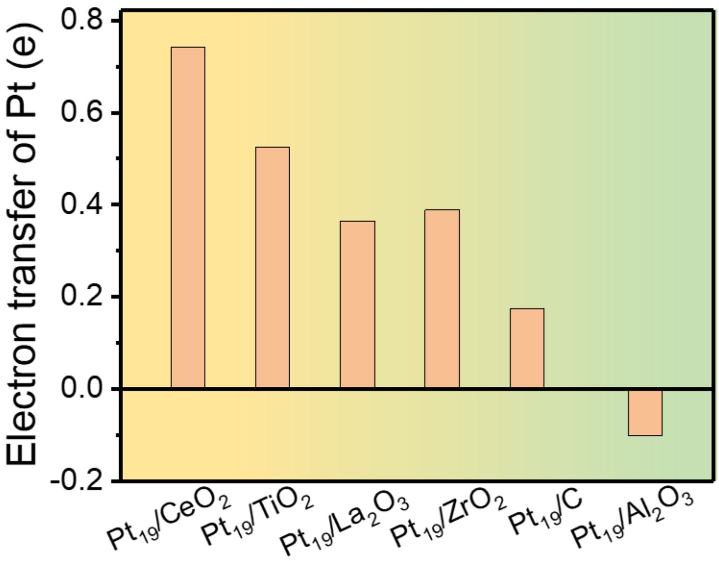


**Supplementary Fig. 11. Bader charge analysis of Pt over varied catalysts.** The electron transfer of $Pt_{19}$ cluster on the representative catalyst models. Positive values represent electron transfer from Pt to carrier while the negative value means the opposite trend.

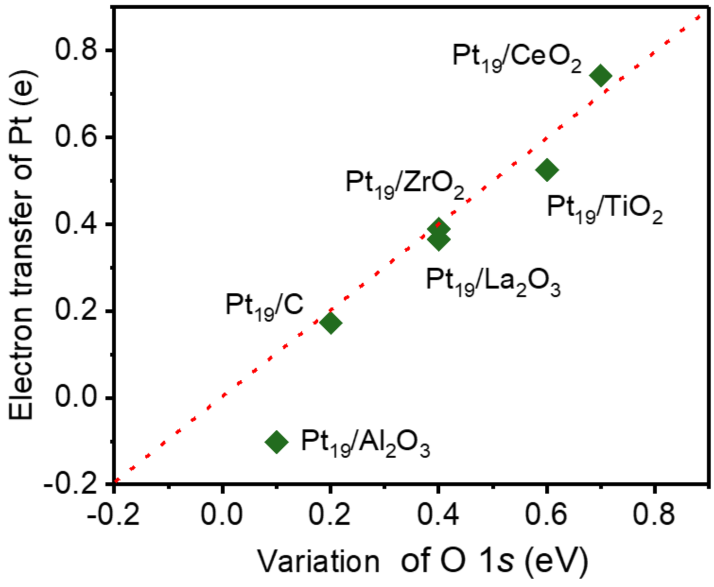


**Supplementary Fig. 12. Analysis about charge transfer between Pt and supports.** The correlation between variation of O 1*s* core level obtained from XPS data and electron transfer of Pt drawn by DFT calculations on corresponding models.

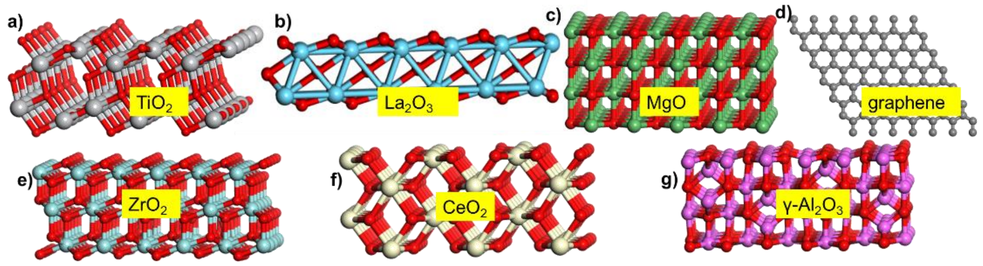


**Supplementary Fig. 13. DFT models of various supports.** The optimized representative models of various support. (a) $TiO_2$, (b) $La_2O_3$, (c) MgO, (d) graphene (denoted as C in the manuscript), (e) $ZrO_2$, (f) $CeO_2$, (g) $\gamma$-$Al_2O_3$.

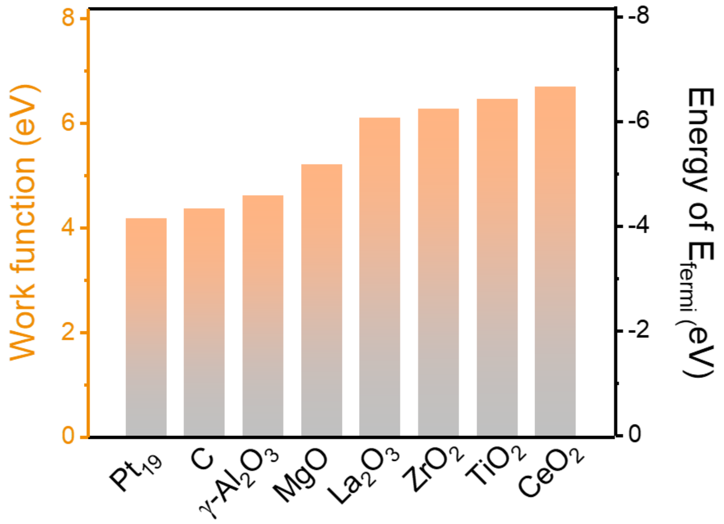


**Supplementary Fig. 14. Work function and fermi level.** The work function and fermi level of $Pt_{19}$ cluster and various support models.

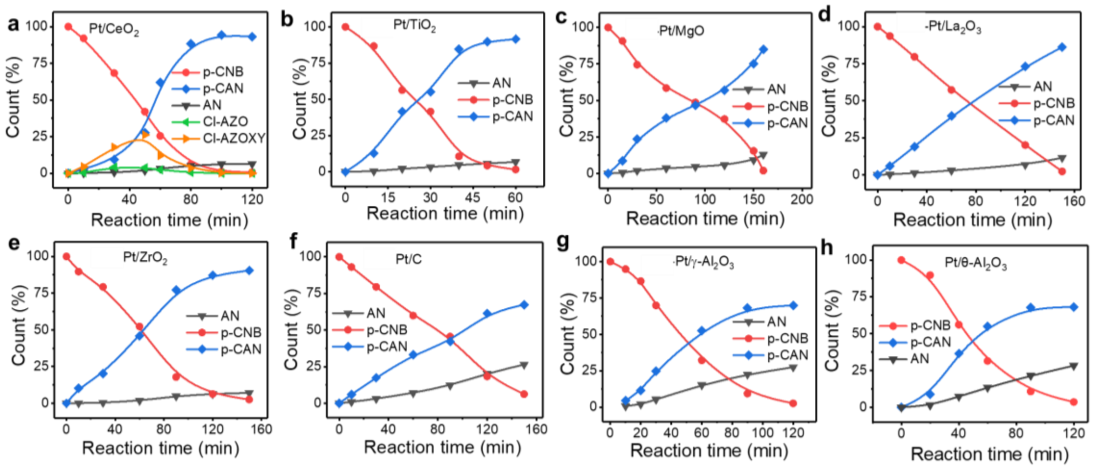


**Supplementary Fig. 15. Distribution of products during the hydrogenation of *p*-CNB over the ~ 2.6nm-based Pt catalysts.** (a) Pt/$CeO_2$. (b) Pt/$TiO_2$. (c) Pt/MgO (d) Pt/$La_2O_3$ (e) Pt/$ZrO_2$, (f) Pt/C, (g) Pt/γ-$Al_2O_3$. (h) Pt/θ-$Al_2O_3$. Reaction conditions: 10 mL toluene, 1 MPa $H_2$, 45 ºC, 0.5 mmol *p*-CNB, Pt/*p*-CNB: 10 wt‰; AN, aniline; *p*-CAN, *p*-chloroaniline; *p*-CNB, *p*-chloronitrobenzene; Cl-AZOXY, Cl-azoxybenzene; Cl-AZO, Cl-azobenzene.

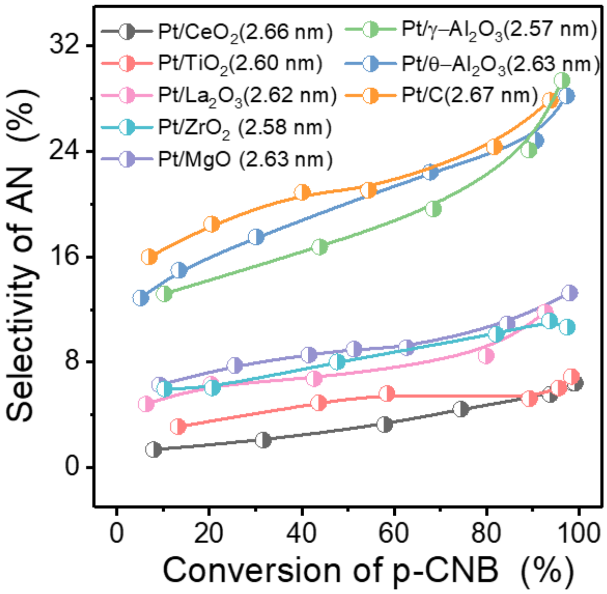


**Supplementary Fig. 16. AN selectivity over varied Pt-based catalysts.** The selectivity to AN as a function of *p*-CNB conversion over varied catalysts with size-controlled Pt NPs in the size range of 2.6 nm. Reaction conditions: 10 mL toluene, 1 MPa $H_2$, 45 °C, 0.5 mmol *p*-CNB, Pt/*p*-CNB: 10 wt‰;

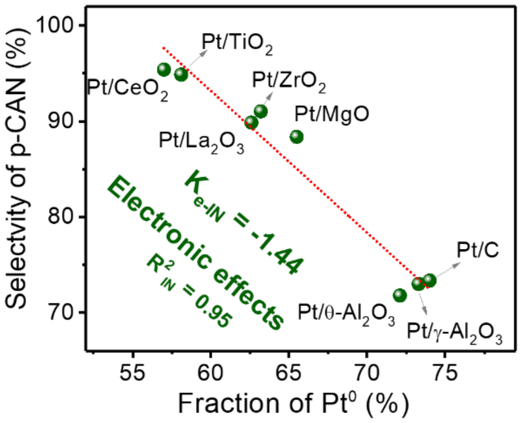


**Supplementary Fig. 17. Relationship between *p*-CAN selectivity and fraction of $Pt^0$ over different catalysts.** Plot of selectivity for *p*-CAN against the fraction of $Pt^0$ on different supported Pt catalysts with the similar size of ~2.6 nm. The data of $Pt^0$ was obtained from the quasi *in-situ* XPS.

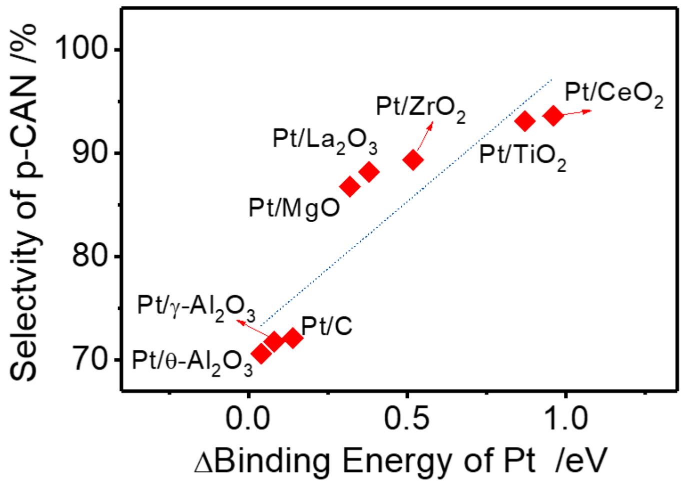


**Supplementary Fig. 18. Relationship between *p*-CAN and ΔB.E$_{Pt}$ over different catalysts.** Plot of *p*-CAN selectivity against the ΔB.E$_{Pt}$ over various catalysts.

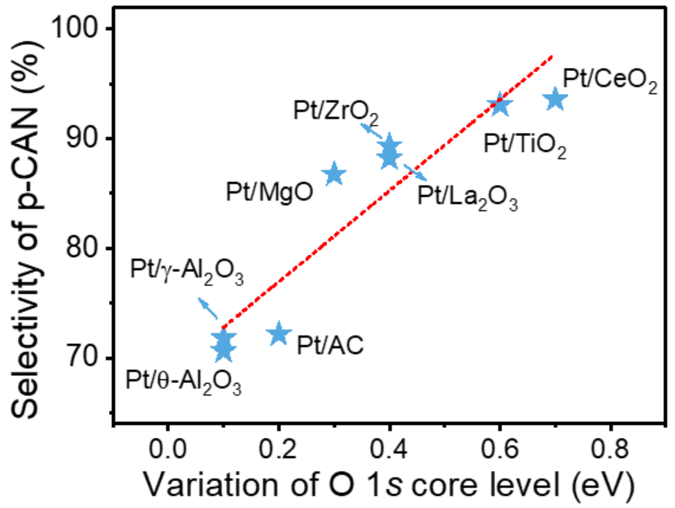


**Supplementary Fig. 19. Relationship between *p*-CAN selectivity and variation of O 1*s*.** Plot of *p*-CAN selectivity against the variation of O 1*s* core level of the support before and after loading the Pt NPs obtained by XPS data.

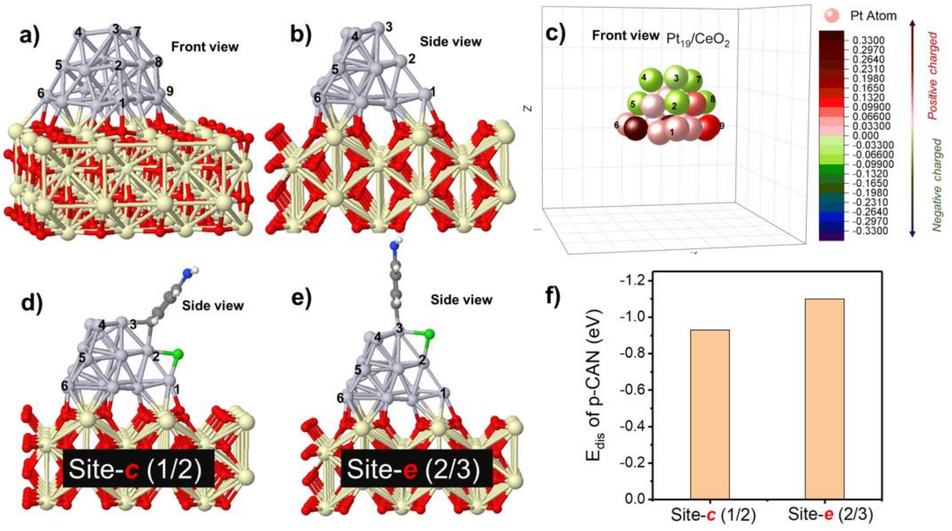

**Supplementary Fig. 20**. **Charge distribution analysis of $Pt_{19}/CeO_2$ derived from DFT calculation.** (a, b) The optimized models of $Pt_{19}/CeO_2$ and (c) the corresponding charge distribution of $Pt_{19}$ NPs. (d, e) Dissociative adsorption configurations of *p*-CAN at different sites and (f) the corresponding $E_{dis}$.

**Supplementary Note 2**: In order to get more insights into the active structure of Pt NPs, as one typical example, bader charge analysis of $Pt_{19}$ clusters on $CeO_2$ had also been done and the results were exhibited as a 3D color map (Supplementary Fig. 20). It is usually considered the metal atoms at bottom layer, directly bonded with support, should display more intense electronic disturbance. And this electronic effect would fade away with increasing the distance between metal atom and support. As indicated in the supplementary figure 20c, it showed that the bottom layer Pt showed more positive charge and these interfacial sites were considered to be the origin of $Pt^{2+}$(Atom 1, 6, 9), as similar with other interfacial sites with partial positive charged metal sites in many literature. And those Pt sites relatively away from $CeO_2$ surface displayed near-neutral charge, representing $Pt^0$ (Atom 3, 4, 7). The dissociation energies ($E_{dis}$) for chemisorption of *p*-CAN, i.e. the dissociation energy of the C-Cl bond in *p*-CAN, were also calculated over varied Pt sites with different electronic properties. It can be seen that the $E_{dis}$ over Site-*c* (–1.09 eV) were higher than that over the electropositive Site-*e* (-0.92 eV). This result suggested that the Pt in the oxidation state are unfavorable for the cleavage of the C-Cl bond, providing the further evidence for our conclusions.

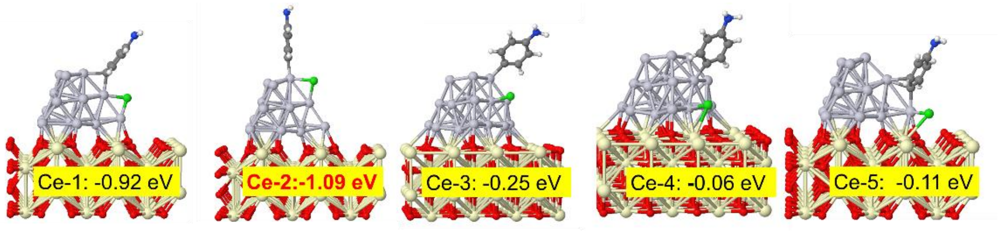


**Supplementary Fig. 21**. **Structure of dissociated *p*-CAN on $Pt_{19}/CeO_2$.** Summary of the optimized dissociative adsorption configurations of *p*-CAN and the corresponding $E_{dis}$ on the $Pt_{19}/CeO_2$ model.

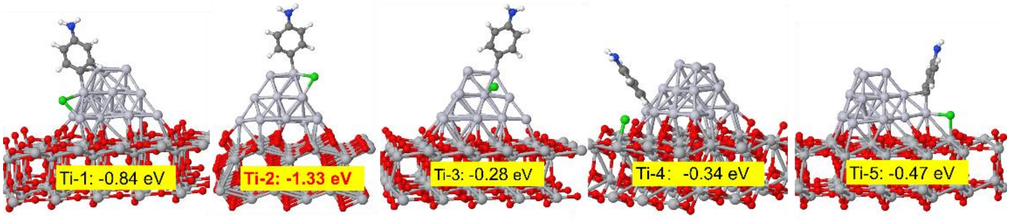


**Supplementary Fig. 22**. **Structure of dissociated *p*-CAN on $Pt_{19}/TiO_2$.** Summary of the optimized dissociative adsorption configurations of *p*-CAN and the corresponding $E_{dis}$ on the $Pt_{19}/TiO_2$ model.

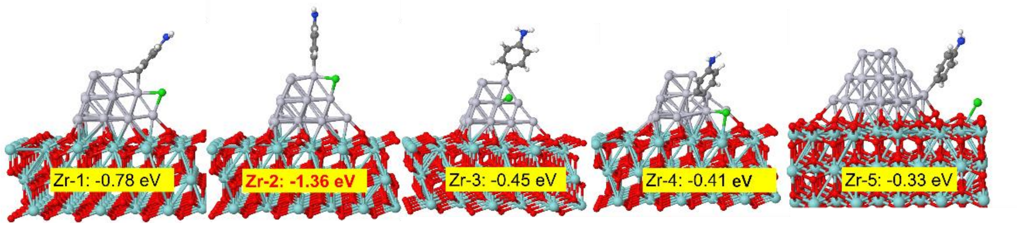


**Supplementary Fig. 23**. **Structure of dissociated *p*-CAN on $Pt_{19}/ZrO_2$.** Summary of the optimized dissociative adsorption configurations of *p*-CAN and the corresponding $E_{dis}$ on the $Pt_{19}/ZrO_2$ model.

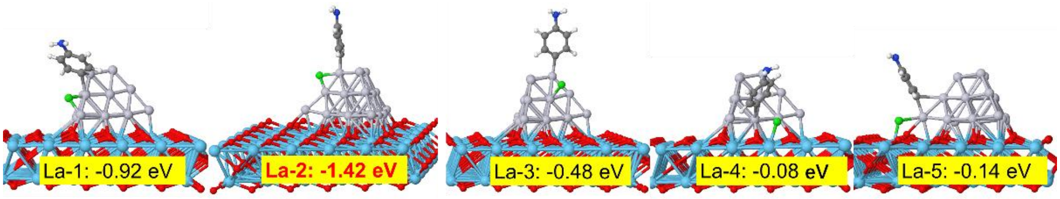


**Supplementary Fig. 24**. **Structure of dissociated *p*-CAN on $Pt_{19}/La_2O_3$.** Summary of the optimized dissociative adsorption configurations of *p*-CAN and the corresponding $E_{dis}$ on the $Pt_{19}/La_2O_3$ model.

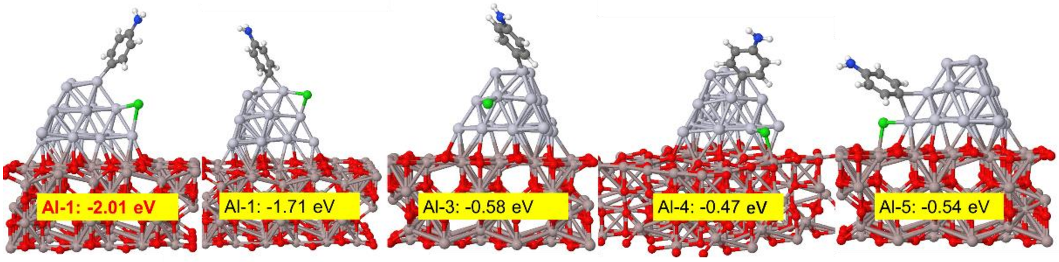


**Supplementary Fig. 25**. **Structure of dissociated *p*-CAN on $Pt_{19}/Al_2O_3$.** Summary of the optimized dissociative adsorption configurations of *p*-CAN and the corresponding $E_{dis}$ on the $Pt_{19}/Al_2O_3$ model.

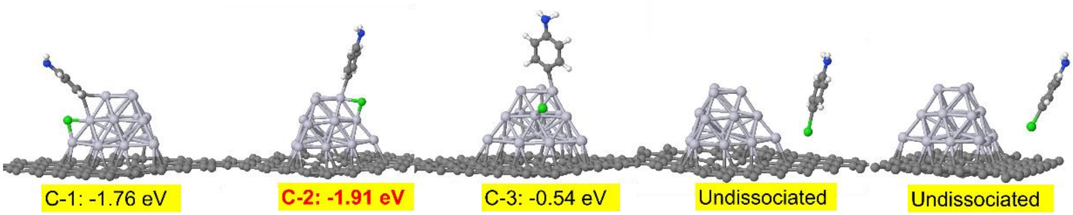


**Supplementary Fig. 26**. **Structure of dissociated *p*-CAN on $Pt_{19}$/C.** Summary of the optimized dissociative adsorption configurations of *p*-CAN and the corresponding $E_{dis}$ on the $Pt_{19}$/C model.

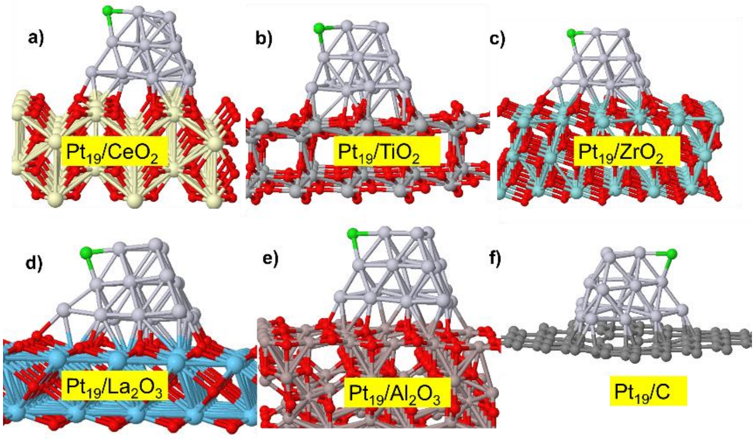


**Supplementary Fig. 27. Structure of adsorbed Cl atom.** Summary of the optimized adsorption configurations of Cl on the representative·Pt-based catalysts models. (a) $Pt_{19}/CeO_2$, (b) $Pt_{19}/TiO_2$, (c) $Pt_{19}/ ZrO_2$, (d) $Pt_{19}/La_2O_3$, (e) $Pt_{19}/ Al_2O_3$. (f) $Pt_{19}/C$.

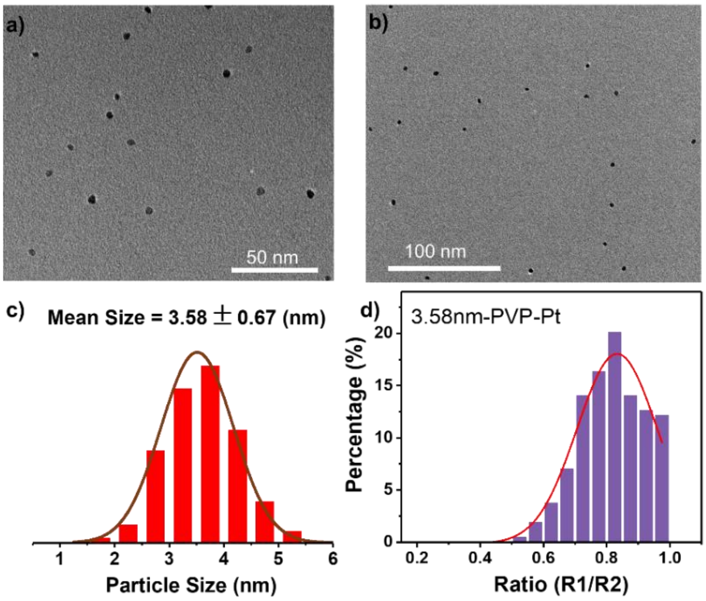


**Supplementary Fig. 28. TEM characterization of 3.58nm-PVP-Pt NPs.** (a, b) TEM images of the PVP-protected Pt particles in the size range of 3.58 nm. (c) Size distribution histograms and (d) the diameter ratio distributions of Pt NPs.

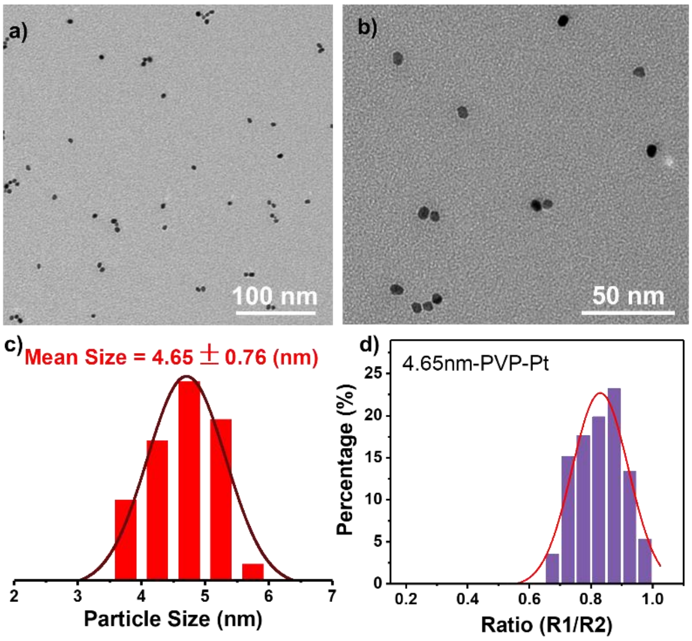


**Supplementary Fig. 29. TEM characterization of 4.65nm-PVP-Pt NPs.** (a, b) TEM images of the PVP-protected Pt particles in the size range of 4.65 nm. (c) Size distribution histograms and (d) the diameter ratio distributions of Pt NPs.

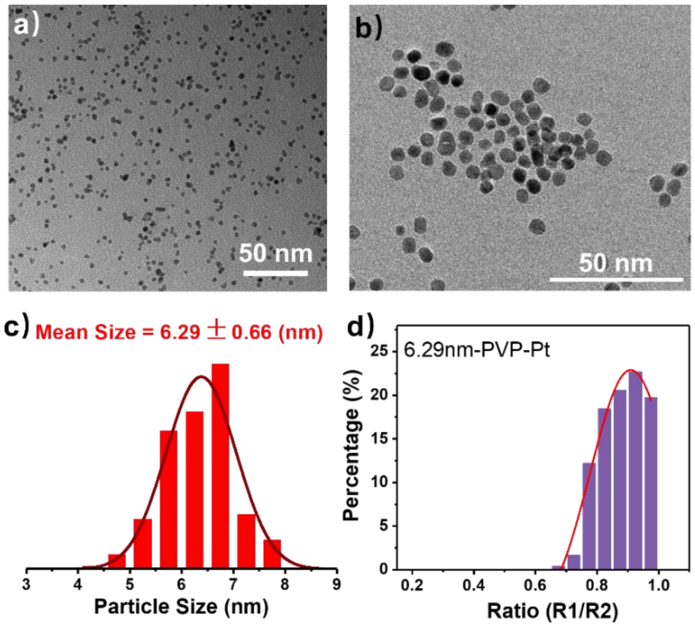


**Supplementary Fig. 30. TEM characterization of 6.29nm-PVP-Pt NPs.** (a, b) TEM images of the PVP-protected Pt particles in the size range of 6.29 nm. (c) Size distribution histograms and (d) the diameter ratio distributions of Pt NPs.

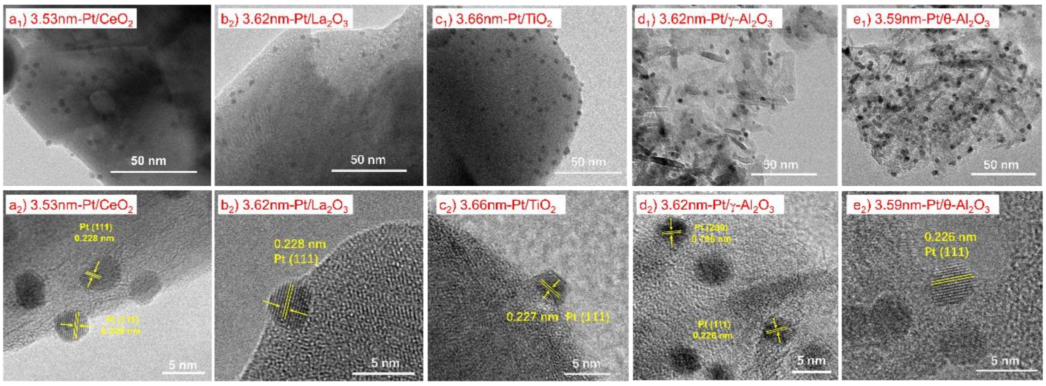


**Supplementary Fig. 31. TEM and HRTEM characterization of series Pt-based catalysts with ~3.6 nm Pt particles.** The representative TEM ($a_1$-$e_1$) and HRTEM ($a_2$-$e_2$) images of as-synthesized catalysts with an average Pt particle size of ~3.6 nm. (a) $Pt/CeO_2$, (b) $Pt/La_2O_3$, (c) $Pt/TiO_2$, (d) $Pt/\gamma\text{-}Al_2O_3$, (e) $Pt/\theta\text{-}Al_2O_3$.

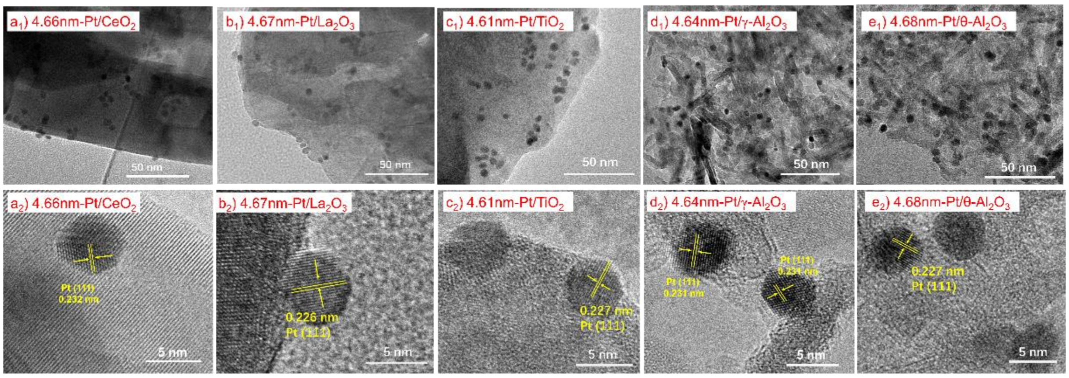


**Supplementary Fig. 32. TEM and HRTEM characterization of series Pt-based catalysts with ~4.6 nm Pt particles.** The representative TEM ($a_1$-$e_1$) and HRTEM ($a_2$-$e_2$) images of as-synthesized catalysts with an average Pt particle size of ~4.6 nm. (a) $Pt/CeO_2$, (b) $Pt/La_2O_3$, (c) $Pt/TiO_2$, (d) $Pt/\gamma\text{-}Al_2O_3$, (e) $Pt/\theta\text{-}Al_2O_3$.

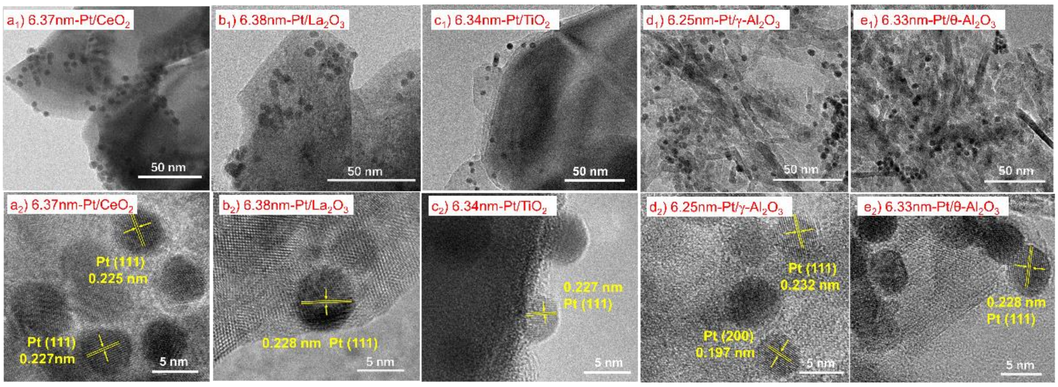


**Supplementary Fig. 33. TEM and HRTEM characterization of series Pt-based catalysts with ~6.3 nm Pt particles.** The representative TEM ($a_1$-$e_1$) and HRTEM ($a_2$-$e_2$) images of as-synthesized catalysts with an average Pt particle size of ~6.3 nm. (a) $Pt/CeO_2$, (b) $Pt/La_2O_3$, (c) $Pt/TiO_2$, (d) $Pt/\gamma$-$Al_2O_3$, (e) $Pt/\theta$-$Al_2O_3$.

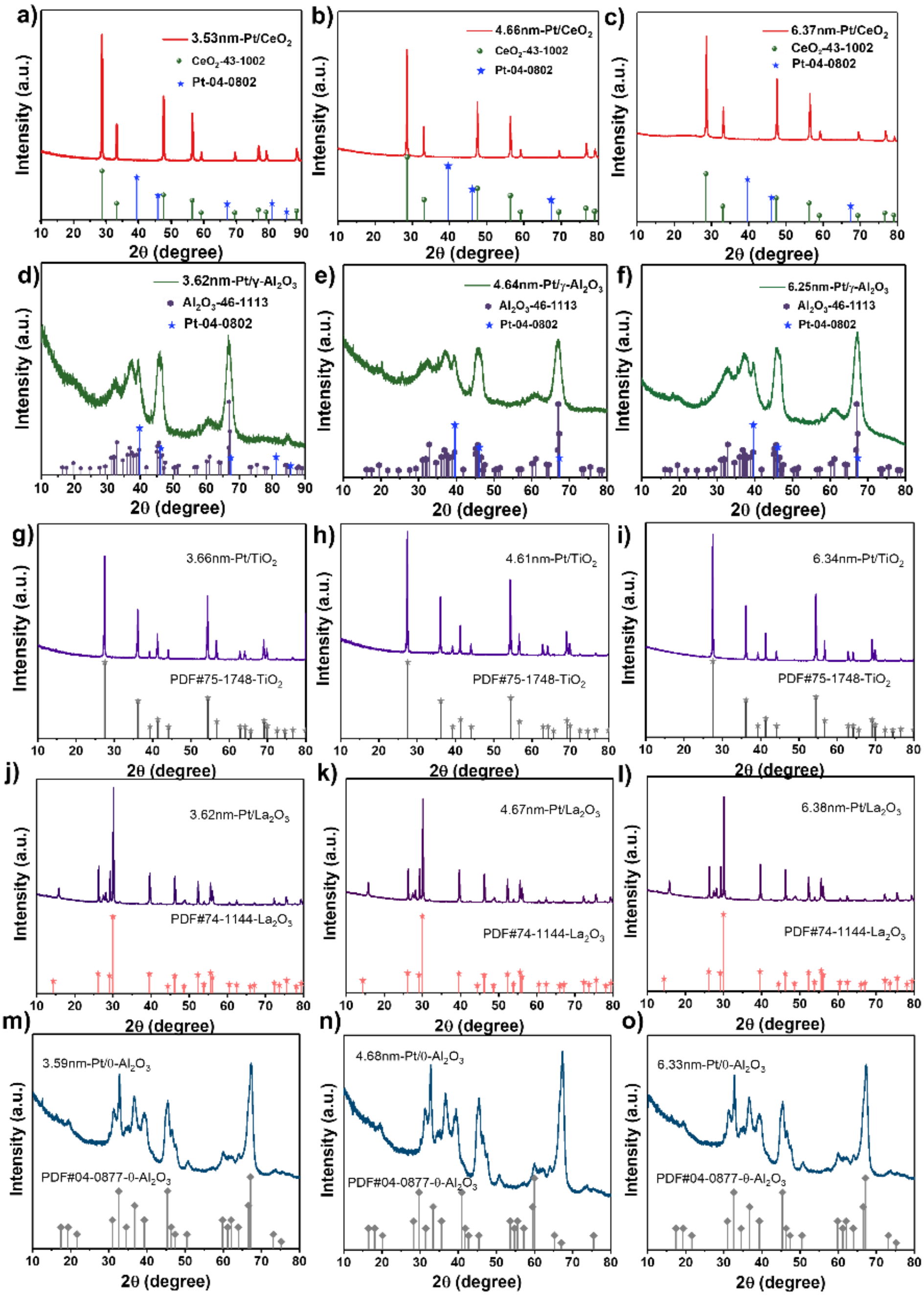


**Supplementary Fig. 34**. **XRD patterns of different catalysts with varied Pt particle size.** (a, b, c) Pt/$CeO_2$, (d, e, f) Pt/γ-$Al_2O_3$, (g, h, i) Pt/$TiO_2$, (j, k, l) Pt/$La_2O_3$, (m, n, o) Pt/θ-$Al_2O_3$.

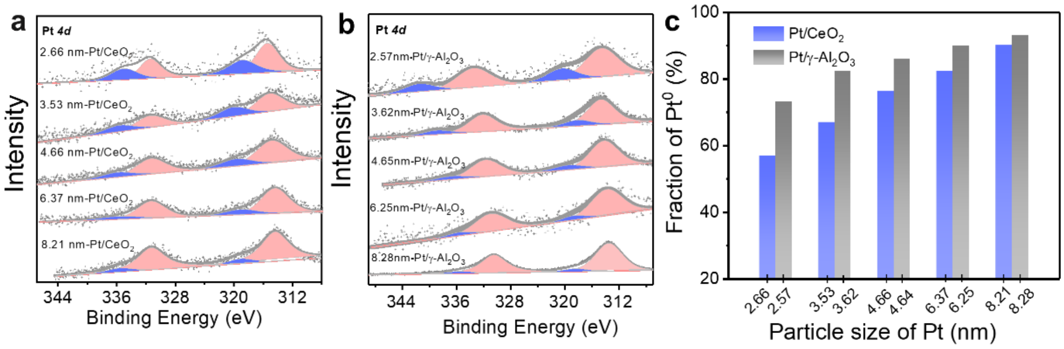


**Supplementary Fig. 35. XPS analysis of series Pt/$CeO_2$ and Pt/γ-$Al_2O_3$ catalysts.** The XPS spectra of the series (a) Pt/$CeO_2$ and (b) Pt/γ-$Al_2O_3$ with varied size in the Pt 4*d* region. (c) Comparison of the fraction of $Pt^0$ in these catalysts.

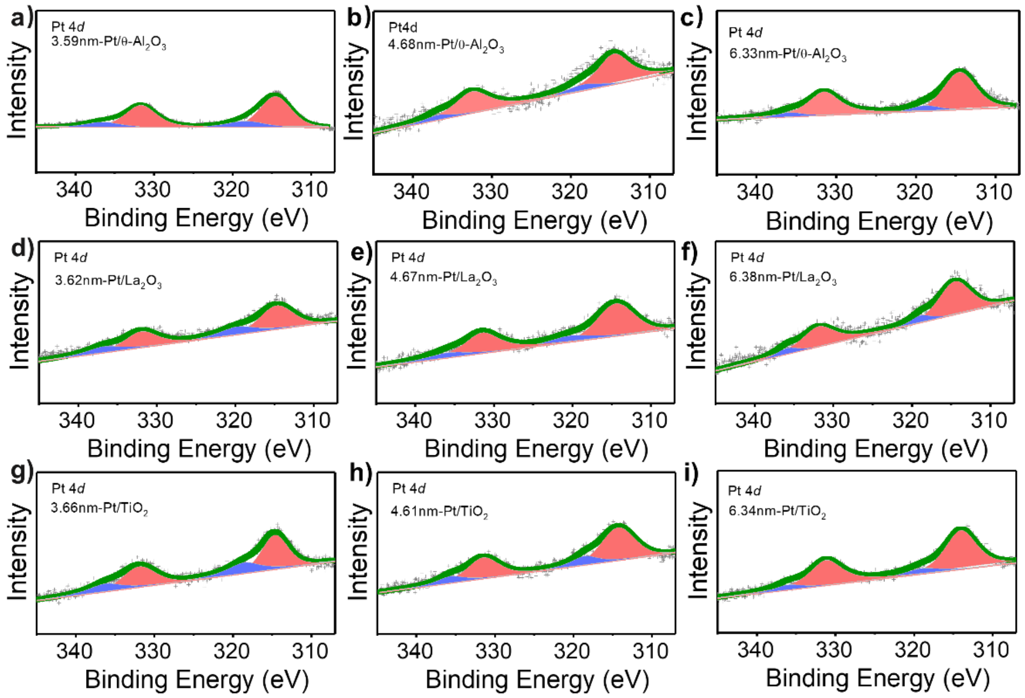


**Supplementary Fig. 36. XPS analysis of varied Pt-based catalysts.** The XPS spectra of the Pt-based catalysts with altered size of Pt in the Pt 4*d* region. (a, b, c) Pt/θ-$Al_2O_3$, (d, e, f) Pt/$La_2O_3$, (g, h, i) Pt/$TiO_2$.

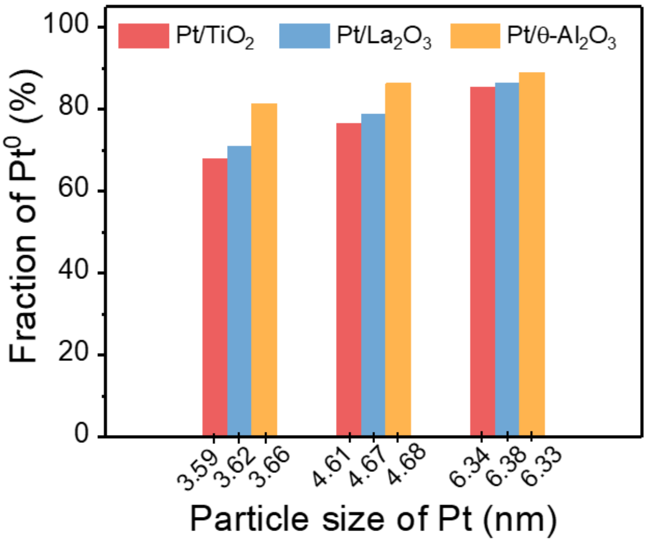


**Supplementary Fig. 37. Analysis of Pt$^0$ content derived from XPS data.** Comparison of the fraction of Pt$^0$ in varied catalysts.

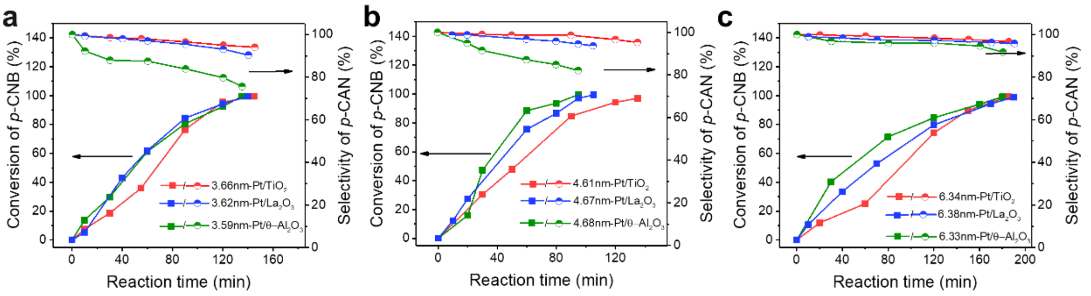


**Supplementary Fig. 38. The plots of *p*-CNB conversion and selectivity of *p*-CAN as a function of reaction time during the hydrogenation of *p*-CNB over different catalysts with varied particle size.** (a) Pt/$TiO_2$, (b) Pt/$La_2O_3$, (c) Pt/θ-$Al_2O_3$. Reaction conditions: 10 mL toluene, 1 MPa $H_2$, 45 ºC, 0.5 mmol *p*-CNB, Pt/*p*-CNB: 10 wt‰; AN, aniline; *p*-CAN, *p*-chloroaniline; *p*-CNB, *p*-chloronitrobenzene;

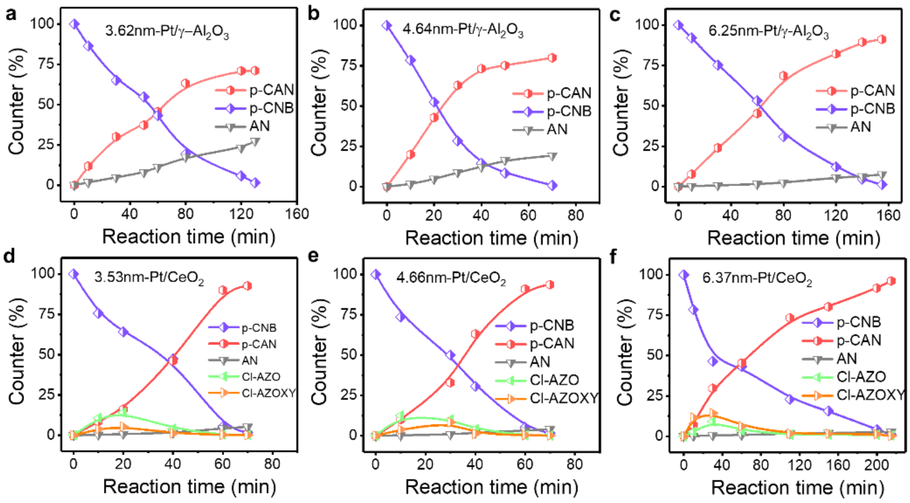


**Supplementary Fig. 39. Distribution of products during the hydrogenation of *p*-CNB over series Pt-based catalysts with altered particle size.** (a, b, c) Pt/γ-$Al_2O_3$, (d, e, f) Pt/$CeO_2$. Reaction conditions: 10 mL toluene, 1 MPa $H_2$, 45 °C, 0.5 mmol *p*-CNB, Pt/*p*-CNB: 10 wt‰; AN, aniline; *p*-CAN, *p*-chloroaniline; *p*-CNB, *p*-chloronitrobenzene; Cl-AZOXY, Cl-azoxybenzene; Cl-AZO, Cl-azobenzene.

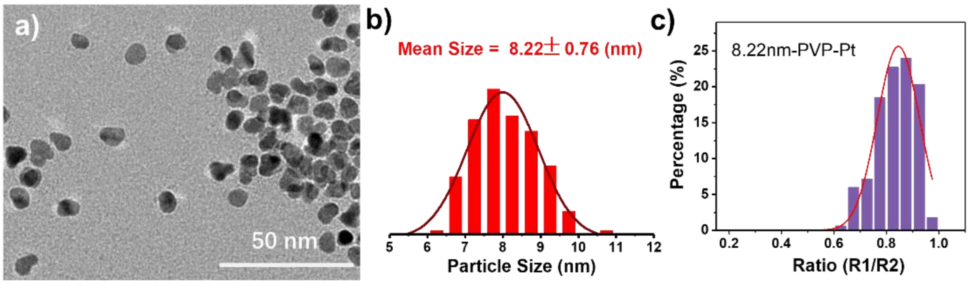


**Supplementary Fig. 40. TEM characterization of 8.22nm-PVP-Pt NPs.** (a) TEM images of the PVP-protected Pt particles in the size range of 8.22 nm. (b) Size distribution histograms and (c) the diameter ratio distributions of Pt NPs.

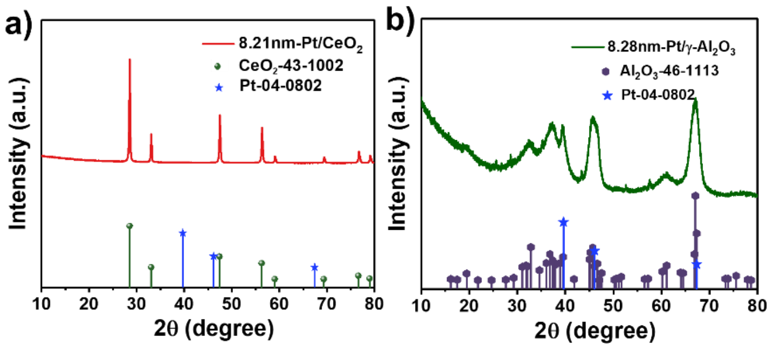


**Supplementary Fig. 41. XRD spectra of different Pt-based catalysts**. XRD patterns of the (a) 8.21nm-Pt/$CeO_2$ and (b) 8.28nm-Pt/γ-$Al_2O_3$.

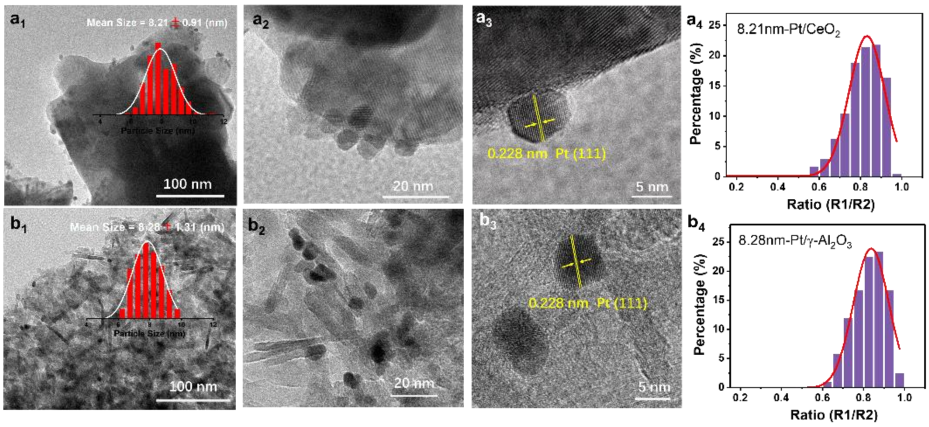


**Supplementary Fig. 42. TEM and HRTEM characterization of 8.21nm-Pt/$CeO_2$ and 8.28nm-Pt/γ-$Al_2O_3$.** TEM, HRTEM images and R1/R2 (a) 8.21nm-Pt/$CeO_2$ and (b) 8.28nm-Pt/γ-$Al_2O_3$. Insert in $a_1$ and $b_1$ were size distribution histograms of Pt NPs.

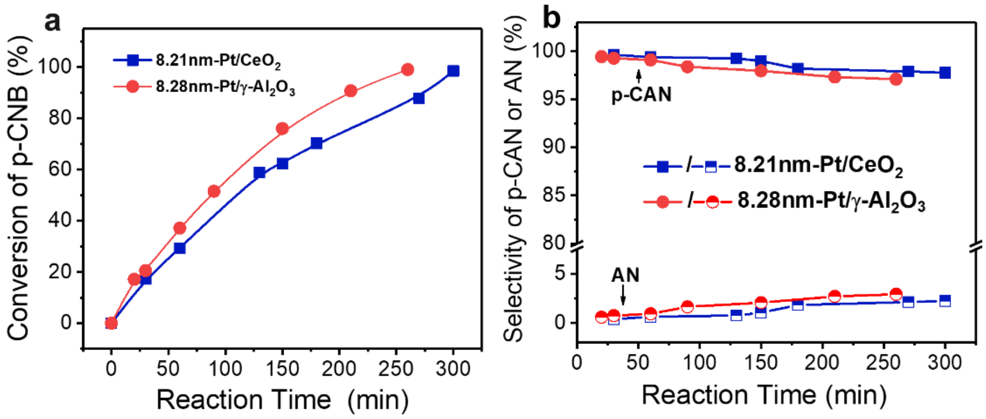


**Supplementary Fig. 43**. **Catalytic performance of 8.21nm-Pt/$CeO_2$ and 8.28nm-Pt/γ-$Al_2O_3$ in chemoselective hydrogenation of *p*-CNB.** (a) The plots of *p*-CNB conversion as a function of reaction time over different catalysts. (b) The selectivity of *p*-CAN and AN as a function of reaction time over different catalysts.

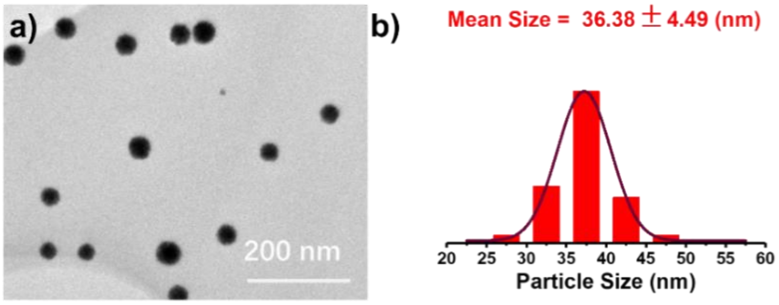


**Supplementary Fig. 44. TEM characterization of 36.38nm-PVP-Pt NPs.** TEM (a) and size distribution histograms (b) of Pt NPs with an average size of ~36 nm.

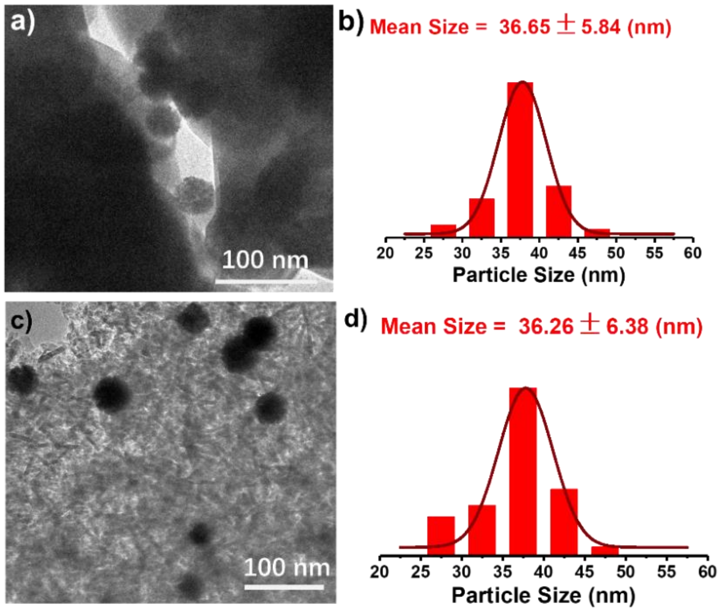


**Supplementary Fig. 45. TEM characterization of 36.65nm-Pt/$CeO_2$ and 36.26nm-Pt/γ-$Al_2O_3$.** TEM iamges and size distribution histograms of (a, b) 36.65nm-Pt/$CeO_2$ and (c, d) 36.26nm-Pt/γ-$Al_2O_3$.

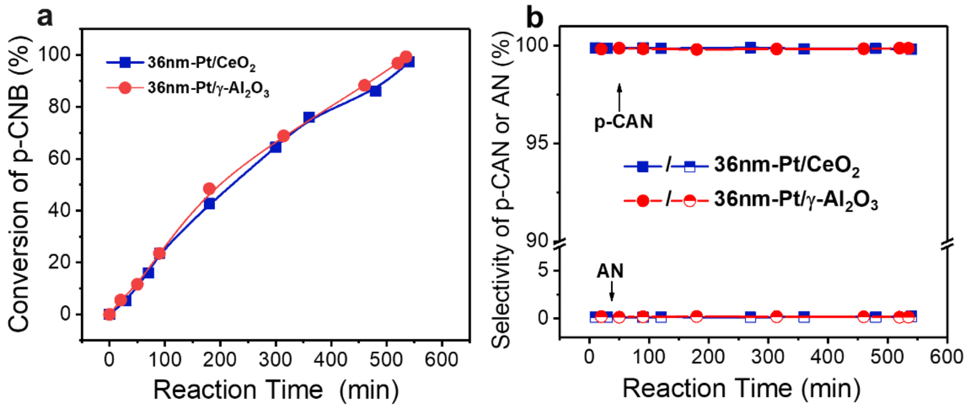


**Supplementary Fig. 46. Catalytic performance of 36.65nm-Pt/$CeO_2$ and 36.26nm-Pt/γ-$Al_2O_3$ in chemoselective hydrogenation of *p*-CNB.** (a) The plots of *p*-CNB conversion as a function of reaction time over different catalysts. (b) The selectivity of *p*-CAN and AN as a function of reaction time over different catalysts.

**Supplementary Table 1.** BET surface area of various supports.

| Support | BET surface area ($m^2$ $g^{-1}$) |
|---|---|
| θ-$Al_2O_3$ | 103.7 |
| γ-$Al_2O_3$ | 164.7 |
| $CeO_2$ | 5.3 |
| $TiO_2$ | 9.1 |
| $ZrO_2$ | 24.9 |
| $La_2O_3$ | 3.6 |
| MgO | 11.9 |
| Pt/C | 1492.2 |

**Supplementary Table 2**. Pt loading weight of the as-synthetized supported Pt catalysts

| Catalysts | Pt weight loading (wt%) |
|---|---|
| Pt/θ-$Al_2O_3$ | 0.34 |
| Pt/γ-$Al_2O_3$ | 0.31 |
| Pt/$CeO_2$ | 0.26 |
| Pt/$TiO_2$ | 0.29 |
| Pt/$ZrO_2$ | 0.34 |
| Pt/$La_2O_3$ | 0.36 |
| Pt/MgO | 0.30 |
| Pt/C | 0.33 |

with the average Pt size of 2.6 nm estimated using an ICP spectrometer.

**Supplementary Table 3**. ΔB.E value of Pt 4*d* (or 4*f*) on various supports

| Catalysts | $\Delta B.E_{Pt}$ (eV) | | Content of $Pt^0$ (%) | |
|---|---|---|---|---|
| | [c]*ex-situ* | [d]quasi *in-situ* | [c]*ex-situ* | [d]quasi *in-situ* |
| [a]$Pt/\gamma\text{-}Al_2O_3$ | 0.08 | 0.07 | 73.1 | 73.3 |
| [a]$Pt/\theta\text{-}Al_2O_3$ | 0.04 | 0.03 | 72.3 | 72.1 |
| [a]Pt/C | 0.14 | 0.17 | 73.7 | 74.0 |
| [a]$Pt/CeO_2$ | 0.96 | 0.95 | 56.8 | 56.6 |
| [a]$Pt/TiO_2$ | 0.87 | 0.88 | 57.7 | 58.0 |
| [a]$Pt/La_2O_3$ | 0.38 | 0.38 | 62.5 | 62.6 |
| [b]Pt/MgO | 0.32 | 0.34 | 64.7 | 65.0 |
| [b]$Pt/ZrO_2$ | 0.52 | 0.51 | 63.3 | 63.1 |

[a]:$\Delta B.E_{Pt} = B.E_{Pt\ 4d\text{-CAT}}$ - 314.5 eV($B.E_{Pt\ 4d\text{-Standard values}}$)

[b]:$\Delta B.E_{Pt} = B.E_{Pt\ 4f\text{-CAT}}$ - 71.0 eV ($B.E_{Pt\ 4f\text{-Standard values}}$)

[c]: the data was obtained from the *ex-situ* XPS spectra of Pt

[d]: the data was obtained from the quasi *in-situ* XPS spectra of Pt

**Supplementary Table 4**. Pt loading weight of the as-synthetized supported Pt catalysts

| Catalysts | Pt weight loading (wt%) |
|---|---|
| 3.62nm-Pt/$\gamma$-$Al_2O_3$ | 0.34 |
| 4.64nm-Pt/$\gamma$-$Al_2O_3$ | 0.36 |
| 6.25nm-Pt/$\gamma$-$Al_2O_3$ | 0.33 |
| 8.28nm-Pt/$\gamma$-$Al_2O_3$ | 0.39 |
| 3.53nm-Pt/$CeO_2$ | 0.34 |
| 4.66nm-Pt/$CeO_2$ | 0.36 |
| 6.37nm-Pt/$CeO_2$ | 0.30 |
| 8.21nm-Pt/$CeO_2$ | 0.28 |
| 3.66nm-Pt/$TiO_2$ | 0.35 |
| 4.61nm-Pt/$TiO_2$ | 0.31 |
| 6.34nm-Pt/$TiO_2$ | 0.36 |
| 3.60nm-Pt/$La_2O_3$ | 0.27 |
| 4.67nm-Pt/$La_2O_3$ | 0.25 |
| 6.38nm-Pt/$La_2O_3$ | 0.22 |
| 3.59nm-Pt/$\theta$-$Al_2O_3$ | 0.41 |
| 4.68nm-Pt/$\theta$-$Al_2O_3$ | 0.31 |
| 6.33nm-Pt/$\theta$-$Al_2O_3$ | 0.36 |

estimated using an ICP spectrometer.
